# ATAC-seq of low-input and cryopreserved primordial germ cells reveals functional enhancers

**DOI:** 10.1242/dev.205214

**Published:** 2026-05-12

**Authors:** Akane Kawaguchi, Mao Igari, Yasuto Murayama, Hiroko Iikawa, Mika Sakamoto, Yasukazu Nakamura, Shigehiro Kuraku, Keisuke Ishihara, Daisuke Saito

**Affiliations:** ^1^ Molecular Life History Laboratory, National Institute of Genetics, Mishima 411-8540, Japan; ^2^ Department of Genetics, Sokendai (Graduate University for Advanced Studies), Mishima 411-8540, Japan; ^3^ Graduate School of Systems Life Sciences, Kyushu University, Fukuoka 819-0395, Japan; ^4^ Chromosome Biochemistry Laboratory, National Institute of Genetics, Mishima 411-8540, Japan; ^5^ Genome Informatics Laboratory, National Institute of Genetics, Mishima 411-8540, Japan; ^6^ Department of Computational and Systems Biology, Pittsburgh, PA 15213, USA; ^7^ Graduate School of Science, Kyushu University, Fukuoka 819-0395, Japan; ^8^ Quantum and Spacetime Research Institute, Kyushu University, Fukuoka 819-0395, Japan

**Keywords:** PGCs enhancers, ATAC-seq, Small input and cryopreserved materials

## Abstract

Dynamic changes in chromatin accessibility at cis-regulatory elements underlie cell fate transitions during development. Primordial germ cells (PGCs) represent a rare population whose chromatin dynamics remain poorly understood compared to known epigenetic landscapes. Here, we utilized the chicken PGC model to bridge this gap, leveraging its capacity for *in vitro* expansion and *in vivo* colonization. We adapted the ATAC-seq workflow to obtain reproducible accessible chromatin region (ACR) profiles from as few as 200 cells, even after cryopreservation. Integrative analysis identified over 10,000 PGC-specific ACRs, many absent from somatic tissues, and revealed inherent Tn5 transposase sequence biases in the chicken genome. To validate these ACRs, we established an *in vitro* PGC differentiation system and utilized *in vivo* embryonic transplantation. Reporter assays confirmed enhancer activities in cultured PGCs, while transcriptome integration associated these ACRs with genes expressed at embryonic day 2.5. *In vivo* transplantation demonstrated that these enhancers exhibited early stage-specific activity, becoming silenced upon gonadal settlement. Our results provide a practical strategy for identifying functional regulatory elements from minimal starting material, facilitating the study of chromatin dynamics in rare cell populations.

## Teaser

**Summary:** A robust strategy for profiling chromatin dynamics in rare cells, uncovering functional enhancers that regulate the fate of avian primordial germ cells and gonadal colonization.

## INTRODUCTION

Cell fate specification and differentiation during development are governed by tightly regulated, dynamic changes in gene expression. Increasing evidence indicates that such transcriptional shifts are orchestrated through changes in chromatin accessibility at cis-regulatory elements. Therefore, capturing the chromatin landscape in a spatiotemporal manner is essential to understand and manipulate lineage decisions.

Primordial germ cells (PGCs) are an inherently small cell population within the developing embryo, yet they carry the unique responsibility of transmitting genomic and epigenetic information to the next generation (Barton et al., 2024; Hansen and Pelegri, 2021; Richardson and Lehmann, 2010). They originate in peripheral regions of the early embryo, and migrate dynamically to the gonads, where they undergo sex-specific programs of mitosis, meiosis, stem cell maintenance and eventual differentiation into gametes (Barton et al., 2024; Wear et al., 2016; Yoshida, 2020). Although DNA and histone modifications in the germline have been extensively studied (Nagano et al., 2022; Seki et al., 2007), how specific genomic regions dynamically open or close during PGC development remains unclear. Furthermore, the high mitochondrial content characteristic of PGCs (Deniz Koc and Yuce, 2012; Motta et al., 2000) presents an inherent technical hurdle for chromatin assays, requiring a robust framework to distinguish nuclear accessibility from mitochondrial contamination.

ATAC-seq (assay for transposase-accessible chromatin using sequencing) enables the genome-wide profiling of accessible chromatin by requiring significantly less input material than the other conventional profiling methods, such as the DNase I hypersensitivity assay (Buenrostro et al., 2013; Ling and Waxman, 2013). The Omni-ATAC-seq protocol further optimized this approach by improving signal-to-noise ratios and minimizing mitochondrial DNA (mtDNA) interference (Corces et al., 2017). However, applying these advancements to rare embryonic populations remains technically demanding, as isolating sufficient number of viable cells is often unfeasible. While cryopreservation offers a potential solution for stabilizing limited samples (Fujiwara et al., 2019; Halstead et al., 2020; Peng et al., 2021), the extent to which cryopreservation distorts chromatin landscapes in low-input regimes has not been rigorously validated. Although single-cell ATAC-seq offers an alternative, its cost and complexity limit its general accessibility (Pott and Lieb, 2015).

In this study, we utilized the unique biological properties of the chicken PGC model to bridge the gap between chromatin accessibility and germline identity. Unlike mammalian models, chicken PGCs can be expanded *in vitro,* while maintaining their full germline potential, including their ability to execute a migratory program and colonize the gonads upon transplantation (Morimoto and Saito, 2025; van de Lavoir et al., 2006; Whyte et al., 2015). Using this system, we established a high-resolution chromatin accessibility map of PGC-specific regulatory landscapes. We evaluated the feasibility of profiling chromatin accessibility in low to high input and live to cryopreserved materials from the *in vitro* cultured chicken PGCs. By refining the OMNI-ATAC-seq workflow, we obtained reproducible accessibility profiles from 200 PGCs, even after freezing. In addition, we also identified potential sequence biases of Tn5 transposase inherent to the chicken genome. To validate the functional relevance of the identified ACRs, we then established an *in vitro* PGC differentiation system for reporter assays and performed embryonic transplantation to confirm enhancer activity *in vitro*. The results demonstrate that our approach can reliably identify functional *cis*-regulatory elements from minimal starting material, providing practical strategies for studying chromatin dynamics in many rare cell populations.

## RESULTS AND DISCUSSION

### Cryopreservation impacts mtDNA content but not overall library quality

**Fig. 1.**
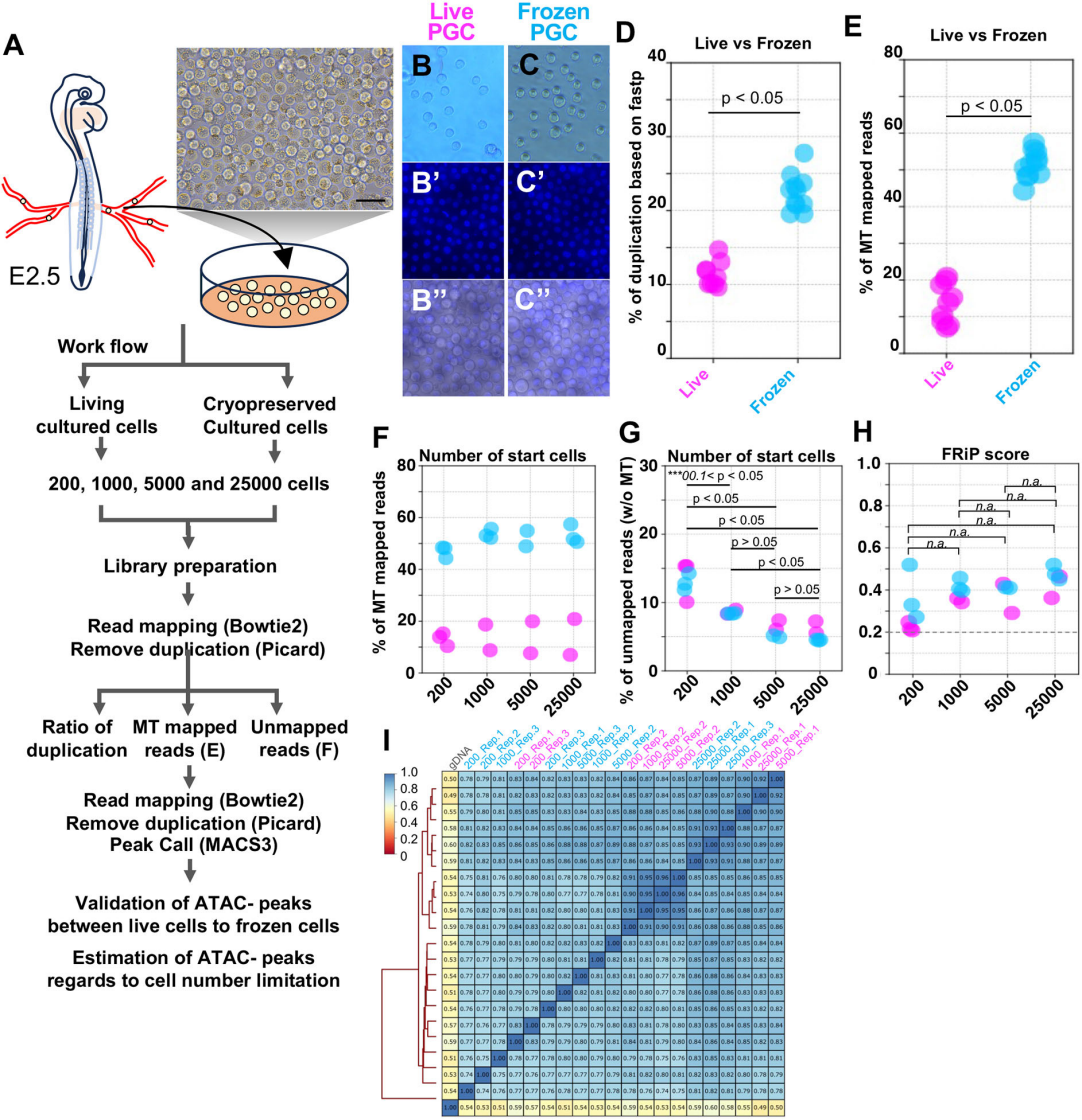
**Quality assessments for Omni-ATAC-seq of live and frozen cultured PGCs.** (A) Schema shows the harvesting of PGCs from E2.5 chicken embryos and the workflow of the Omni-ATAC-seq benchmarking setup. Scale bar: 25 μm. (B-C″) Cell viability analysis using NucBlue staining in live PGCs (B-B″) and frozen PGCs (C-C″). Frozen PGCs retained nuclear morphology comparable to that of live cells. (D-H) Quality metrics across input conditions: (D) duplication rates detected after initial quality control and adapter trimming using Fastp; (E,F) contamination rates of mitochondrial reads; (G) proportion of unmapped reads after alignment; and (H) FRiP score for the individual libraries. In E to I, live and frozen samples are marked with magenta and blue, respectively. (I) Heatmap comparing all Omni-ATAC-seq libraries across a range of input cell numbers for both live and frozen conditions. Statistical analysis was carried out using the Mann-Whitney U-test between each condition and cell number group.

To evaluate the impact of cell number and cryopreservation on Omni-ATAC-seq quality, we prepared cultured chicken PGC samples with four different input sizes (200, 1000, 5000 and 25,000 cells), using either freshly harvested or cryopreserved cells stored at −80°C for 1 week (Fig. 1A). The protocol followed the original Omni-ATAC-seq method (Corces et al., 2017), which employs a combination of detergents (Tween-20, NP-40 and digitonin) for effective membrane permeabilization, and PBS addition during tagmentation to enhance the signal-to-noise ratio by reducing mtDNA contamination. Cryopreserved samples exhibited high cell viability (>90%) based on Trypan Blue exclusion when the cells were stained at the same time with thawing, and nuclear morphology remained comparable to that of live cells, as assessed by nuclear staining (Fig. 1B,C). These results indicate that both fresh and cryopreserved cells were suitable as starting materials. As a negative control, we included a sample of purified genomic DNA subjected to tagmentation. After library construction and sequencing, we processed fastq files through standard pre-processing steps, including adapter trimming, filtering, genome alignment and duplicate removal. Cryopreserved samples exhibited higher duplication rates (19-27%) compared to live cells (9-14%) (Fig. 1D, Table S1), suggesting that freeze-thaw steps may increase redundant amplification.

We next evaluated three key quality metrics of ATAC-seq data: (1) mtDNA (mtDNA) mapping rate, (2) unmapped read rate and (3) FRiP (fraction of reads in peaks) score. The mtDNA read contamination was notably higher in cryopreserved samples (50-60%) than in fresh samples (5-15%) (Fig. 1E,F), suggesting that cryopreservation may reduce the efficiency of mitochondrial removal during lysis. This observation is consistent with earlier reports on ATAC-seq protocols lacking detergent-based optimization (Fujiwara et al., 2019). The proportion of unmapped reads was highest in 200-cell input samples (10-15%), regardless of cryopreservation, while samples with ≥1000 cells showed stable mapping rates (∼5-7%) (Fig. 1G). This suggests that input cell number, rather than freeze-thaw condition, is the major determinant of read mappability. FRiP scores, a standard measure of ATAC-seq signal quality, exceeded 0.2 in all libraries (Fig. 1H), thereby meeting ENCODE guidelines (https://www.encodeproject.org/atac-seq/). While FRiP scores showed a weak correlation with input cell number, they were not significantly influenced by cryopreservation. After removal of duplicate and mtDNA reads, overall correlation patterns were comparable across conditions (Fig. 1I). Although we did not test the mechanism directly, freeze-thaw cycles may alter membrane properties (e.g. lipid composition and/or membrane integrity), reducing the efficiency of detergent-based lysis (Tween-20, digitonin and NP-40) in separating nuclei from mitochondria during the Omni-ATAC workflow. Because mtDNA lacks nucleosomal protection, it is preferentially tagmented by Tn5, leading to decreased library complexity and a higher duplication rate. While this explanation remains to be confirmed, future studies are needed to evaluate membrane-related changes and optimize lysis conditions for cryopreserved materials. Taken together, these results demonstrate that while cryopreservation increases mtDNA contamination and duplication rates, it does not substantially compromise library quality, even in PGC samples, which are known to contain relatively high levels of mtDNA (Deniz Koc and Yuce, 2012; Motta et al., 2000). Nevertheless, reproducible and high-quality chromatin accessibility profiles can be obtained from as few as 200 cells, even after cryopreservation.

### Peak number and sensitivity in low-input cryopreserved samples

**Fig. 2.**
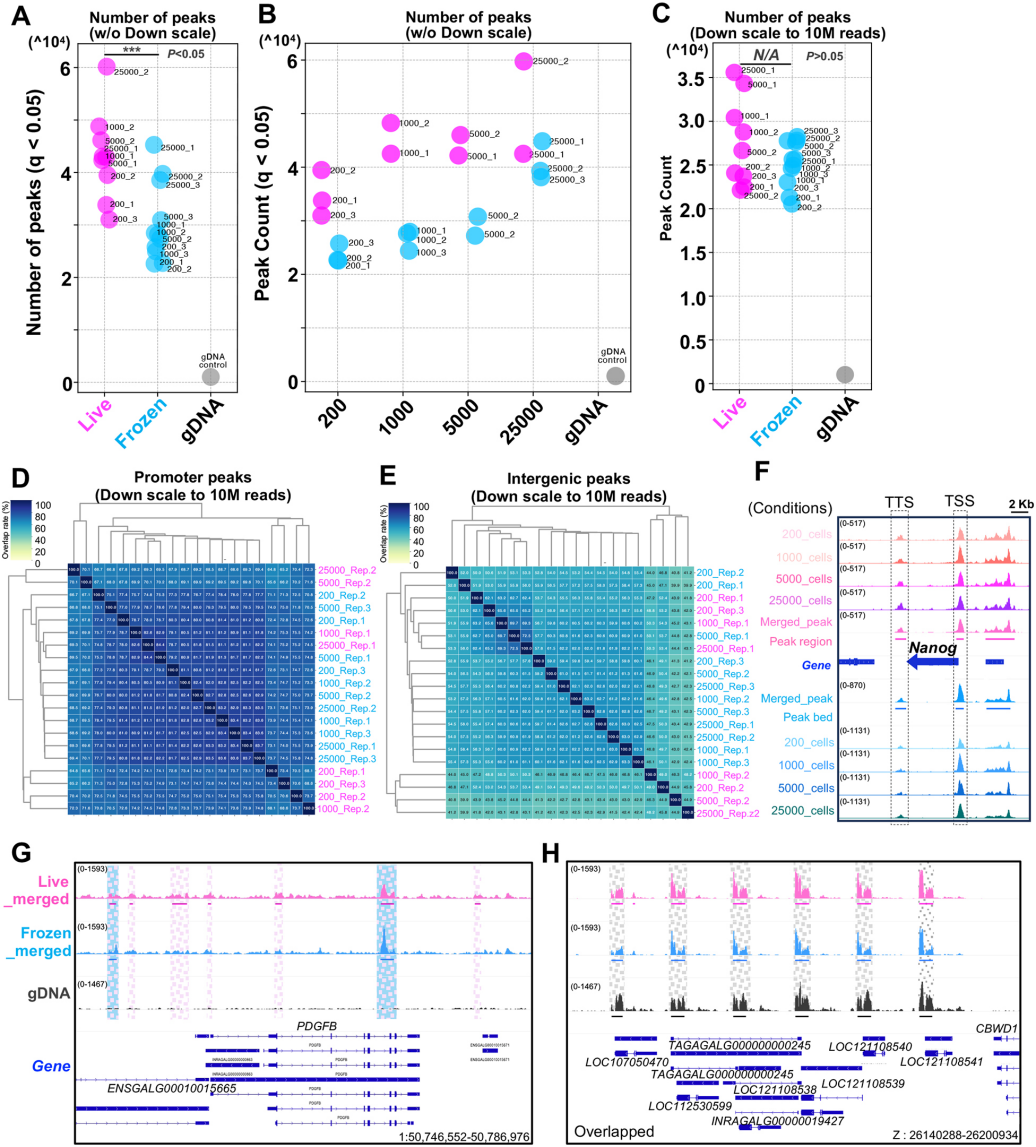
**Omni-ATAC-seq from frozen PGCs reduced sensitivity for weak peaks in cryopreserved samples, whereas live and frozen samples still exhibit consistent promoter accessibility at strong peaks.** (A) Number of peaks detected by MACS3 (--broad, --q 0.05) without read downscaling, across input sizes and preservation conditions. Peak numbers were significantly lower in frozen samples (blue) compared to live samples (magenta) (unpaired two-tailed *t*-test, ***P<0.05). (B) Relationship between input cell number and peak counts (without downscaling). A slight increases in peak counts were observed with higher input, regardless of preservation. (C) Number of peaks detected after normalizing all libraries to 10 million valid reads using scaling factors (no significant difference, *P*>0.05; Mann-Whitney U-test (unpaired, two-tailed). (D,E) Heatmaps showing pairwise peak overlaps after downscaling, for promoter-associated peaks (D) and intergenic peaks (E). Promoter peaks were highly concordant across all conditions, while intergenic peaks exhibited greater variability. (F) IGV tracks at *Nanog* locus show consistent peak detection across conditions and input levels at the transcription start site (TSS) and transcription termination site (TTS), with merged replicates enhancing signal clarity. (G) Representative genome browser views showing accessible chromatin regions in live, frozen and gDNA control samples. Live-specific low-intensity peaks are reduced or absent in frozen and gDNA samples. (H) Representative genome browser shows that frozen PGC ATAC-seq libraries fail to detect weak ACRs visible in live samples. Peaks in gDNA samples are enriched at interstitial telomeric sequences (ITSs), reflecting sequence preference of the Tn5 transposase.

We next examined how variations in input cell number and cryopreservation influence the number and quality of peaks detected by ATAC-seq. To begin, peak calling was performed using MACS3 without normalization (–broad, *q*<0.05). In live-cell libraries, the number of identified peaks ranged from 31,018 to 48,786. In contrast, cryopreserved libraries yielded significantly fewer peaks, between 22,737 and 38,558, with a statistically significant reduction compared to live samples (*P*<0.05, Fig. 2A, Table S1). Peak counts showed a modest upward trend, with increasing input cell numbers in both conditions (*P*<0.05, Fig. 2B).

To assess whether these differences stemmed from sequencing depth, we next normalized all libraries to 10 million valid reads using computed scaling factors (Table S1). After reading subsampling and re-calling peaks with identical MACS3 parameters, peak numbers across all libraries converged, with no statistically significant differences between groups (*P*>0.05), except for the gDNA-derived negative control (Fig. 2C). It suggests that the reduced peak counts in cryopreserved samples primarily reflect lower sequencing depth rather than intrinsic biological differences.

We further assessed peak reproducibility by annotating all ATAC-seq peaks to genomic features (promoter, exon, intron, intergenic and transcription termination site regions) and computing overlap rates. Promoter-associated peaks exhibited the highest reproducibility across all samples, with overlap rates of 70-85%, regardless of preservation method or cell number (Fig. 2D). Peaks located in intergenic, intronic, exonic and TTS regions were more variable (∼65% overlap), likely due to the weaker and more heterogeneous nature of regulatory elements in these regions (Fig. 2E, Fig. S1A-C).

To evaluate signal robustness at key regulatory loci, we visualized the detected ACRs at the *Nanog* promoter, a well-characterized marker of PGC identity and pluripotency (Choi et al., 2021). Consistent peaks were observed across all conditions, including cryopreserved 200-cell samples (Fig. 2F). To determine whether increased sequencing depth could restore detection of these weak peaks, we merged reads across samples from the same condition (treated as biological replicates) after removing mitochondrial and PCR-duplicated reads (Fig. 2G). The merged datasets effectively recovered some of the weakly accessible regions, suggesting that deeper sequencing can mitigate the sensitivity loss associated with cryopreservation.

We also investigated potential sequence bias introduced by the Tn5 transposase using a gDNA control library as a ‘blacklist’. Peak calling and motif enrichment analysis of this control yielded 1023 peaks, disproportionately enriched in intergenic regions containing the telomeric (TTAGGG)n hexamer, likely corresponding to interstitial telomeric sequences (ITSs) (Bolzan, 2017) (Fig. 2H, Fig. S1D-J). The chicken genome is characterized by a distinctive karyotype comprising macro- and micro- chromosomes, and ITSs have been recognized as genomic signatures shaped by meiotic recombination and chromosomal rearrangements throughout its evolutionary history (Nanda et al., 1995). Assessing sequence bias using this gDNA-negative control provides a valuable means to distinguish genuine biological signals from technical artifacts reflecting inherent sequence preferences of Tn5 transposase. Our results demonstrated that, while Omni-ATAC-seq applied to low-input frozen samples yields chromatin accessibility profiles largely comparable to those of live cells, incorporating a gDNA-based strategy is especially relevant for species with distinctive genomic architectures. This approach ensures more accurate identification of regulatory elements and expands the applicability of Omni-ATAC-seq to a wider range of rare or previously inaccessible cell populations.

### Identification of PGC-specific accessible chromatin regions

**Fig. 3.**
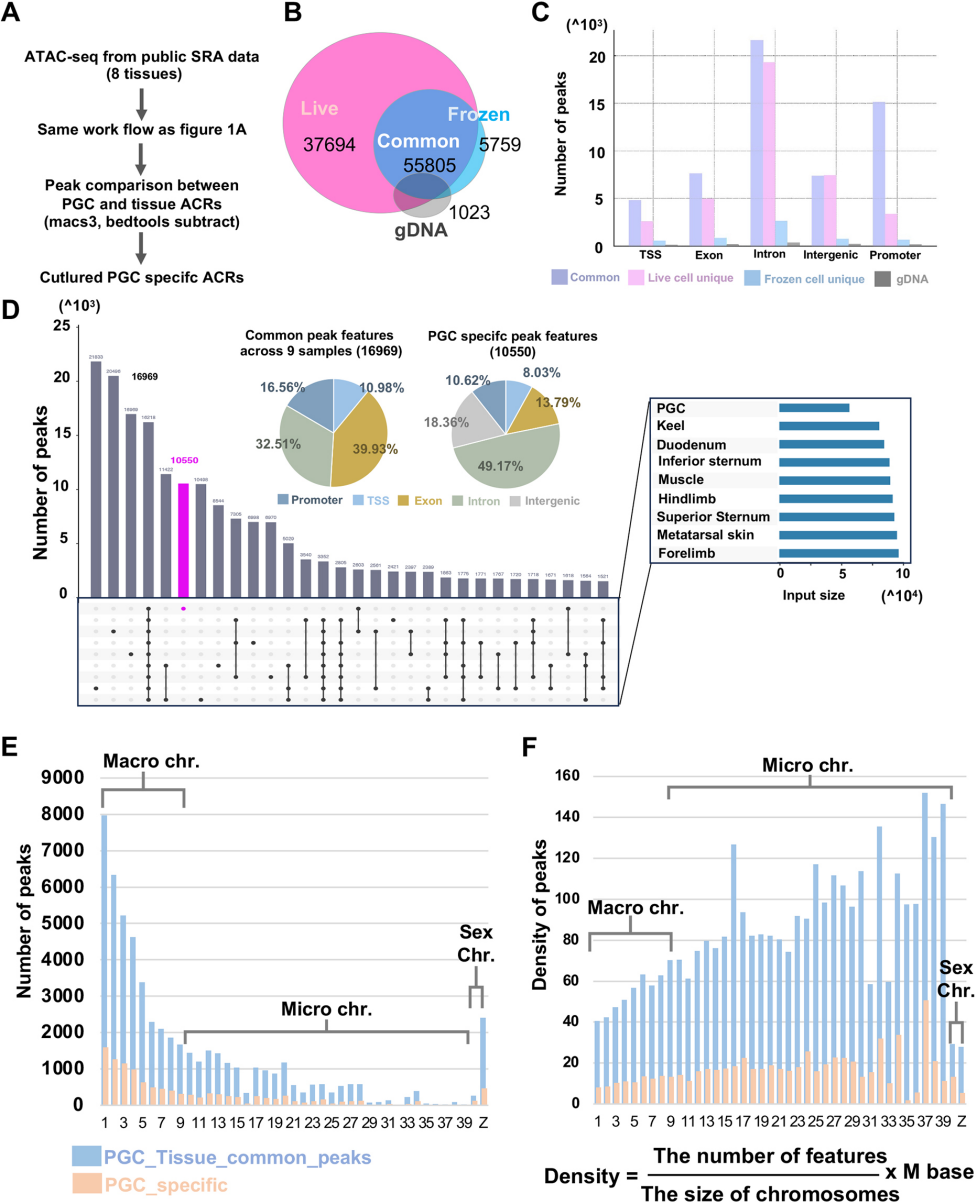
**Comparison of PGC and somatic tissue ATAC-seq samples enabled the identification of cell-type-specific cis-regulatory elements and their distribution across chromosomes.** (A) Schema of the analysis pipeline: ACRs identified in cultured PGCs were compared with published ATAC-seq datasets from somatic tissues to define PGC-specific ACRs. (B) Venn diagram showing the number of peaks detected in live and cryopreserved PGCs, and their overlap. A total of 55,805 peaks were shared between conditions, with live samples contributing a greater number of unique peaks. (C) Peak annotation of ACRs categorized by feature (promoter, exon, intron, intergenic and TTS). Many populations of intronic and intergenic peaks were detected and biased in live samples. (D) Upset plot illustrating peak overlaps among eight somatic tissue datasets. Bar plots indicate total peak numbers; pie charts show genomic feature distributions of commonly shared ACRs (left) and PGC-specific ACRs (right). (E) The counts of PGC-specific ACRs per chromosome. (F) Peak densities normalized by chromosome length (peaks per Mbp). Common ACRs are marked in blue and PGC-specific ACRs are in pink.

We next asked whether it is possible to identify functional ACRs that show either PGC-specific or developmental stage-specific accessibility, by integrative analysis with publicly available ATAC-seq datasets (Fig. 3A). To identify PGC-specific regulatory elements, instead of differential peak analysis we applied a binary comparison strategy, subtracting peaks shared with multiple somatic tissues to remove commonly accessible ACRs (Fig. 3A). We merged our datasets by input type (live or cryopreserved), each yielding 195-247 million mapped reads, so that the library volume became comparable and robust to other tissue libraries (Table S2). Peak calling produced 55,805 overlapping peaks between live and cryopreserved PGCs (Fig. 3B). Live-specific peaks (∼30,000) were far more numerous than cryopreserved-specific peaks (∼5759), with shared peaks mainly in promoter regions and live-specific peaks enriched in intronic and intergenic regions, and the observed genomic distribution is consistent with the previous results in Fig. 2 (Fig. 3C, Tables S3-S6).

To define PGC-specific ACRs, we compared peaks shared by live and cryopreserved samples with a binary matrix from 30 public ATAC-seq datasets of eight somatic tissues and embryonic organs (Fig. 3A). This comparison resulted in 10,550 PGC-specific ACRs (Fig. 3D, Table S4), distributed across promoters (10.62%), exons (13.79%), introns (49.17%), intergenic (18.36%) and TTSs (8.03%). In contrast, 16,969 ACRs shared with somatic tissues were enriched in promoters (16.56%) and exons (39.93%), with no intergenic contribution. Overall, ACR numbers in PGCs were comparable to somatic tissues. These findings suggest that PGC-specific expression is largely controlled by enhancers in non-coding regions, rather than promoter-proximal sites, and further indicate that intergenic ACRs at housekeeping genes are not uniformly accessible across tissues or cell types. Furthermore, we characterized the chromosomal distribution of PGC-specific ACRs to identify potential genomic structural bias across macro- and micro-chromosomes (Fig. 3E, Fig. S2). When normalized by chromosome length (Mbp), the density of PGC-specific ACRs was slightly lower on macro-chromosomes and higher on micro-chromosomes (Fig. 3D), a pattern mirroring the underlying density of protein-coding genes and non-coding RNAs (lncRNA, miRNA, ncRNAs and others) (Fig. S2B). Notably, the sex chromosomes exhibited distinct profiles. The density of PGC-specific ACRs on the W chromosome (13.39/Mbp) was substantially higher than on the Z chromosome (5.39/Mbp). This suggests potential sex chromosome-associated regulatory features unique to PGCs, warranting further investigation into sex-dependent PGC epigenetic regulations in these cells.

To identify functional PGC-specific enhancers, we implemented two complementary selection strategies designed to balance stringency and reproducibility (Table S7). First, the top 16 ACRs from frozen 200-cell libraries (F200-series) were selected based on fold-change and statistical significance. To maximize stringency under low-input conditions, we required reproducible peaks across three independent 200-cell replicates and excluded ACRs detected in somatic tissue by binary subtraction (the genomic coordinates and meanFold values of F-200 series are provided in Table S8). Second, ACRs detected in both live and cryopreserved libraries were defined as the common set (C-series). From this set, the top 19 ACRs were selected based on fold-change and statistical significance as high-confidence regulatory elements.

To prioritize active enhancers rather than merely open chromatin, we intersected PGC-specific ACRs with H3K27ac CUT&Tag profiles from cultured PGCs and identified 675 H3K27ac-positive PGC-specific ACRs (all the detected 11,339 of H3K27ac regions and values are listed in Table S9). Among the 31 reported candidates subjected to functional testing, six exhibited clear H3K27ac enrichment (Table S7). Given the rapid turnover of histone acetylation, this likely represents a conservative estimate of enhancer activation, consistent with the dynamic and state-dependent nature of active regulatory elements (Creyghton et al., 2010; Kaya-Okur et al., 2019).

### Functional validation of candidate PGC-specific ACRs in cultured cells

To validate enhancer activity, we performed a fluorescence-based reporter assay in cultured PGCs. Candidate ACRs were cloned upstream of a minimal tk promoter driving EGFP (Uchikawa et al., 2003) and co-transfected with a CAGGS-mCherry plasmid as an internal control for transfection efficiency and cell recovery (Fig. S3).

We first examine F200 candidates derived from cryopreserved 200-cell libraries. Sixteen top-ranked regions were selected, of which 14 were successfully cloned (Tables S7 and S8). Eleven (F200-2, −4, −5, −7, −8, −9, −10, −11, −13, −14 and −16) of these 14 constructs drove EGFP expression above basal levels in cultured PGCs (Fig. S3), with several exhibiting activity comparable to the Nanog promoter. mCherry fluorescence was detected in all samples. The active constructs corresponded to strong accessibility peaks in the ATAC-seq dataset (Fig. S4).

We next tested candidates from the common set (C-series) (Table S4). After excluding four regions (C-4, −11, −13 and −18) overlapping with F200 candidates (corresponding to F200-7, −5, −4 and −14), eight (C-1, −3, −8, −12, −15, −16, −17 and −19) of the remaining 15 constructs were successfully cloned and all showed enhancer activity in cultured PGCs (Fig. S5). These regions also displayed robust accessibility signals in the ATAC-seq dataset.

Collectively, these results demonstrate that PGC-specific ACRs identified from cryopreserved 200-cell libraries are functionally equivalent to those derived from the common dataset, confirming that biologically meaningful enhancers can be recovered even under minimal-input and cryopreserved conditions.

### Loss of enhancer activity upon PGC differentiation

**Fig. 4.**
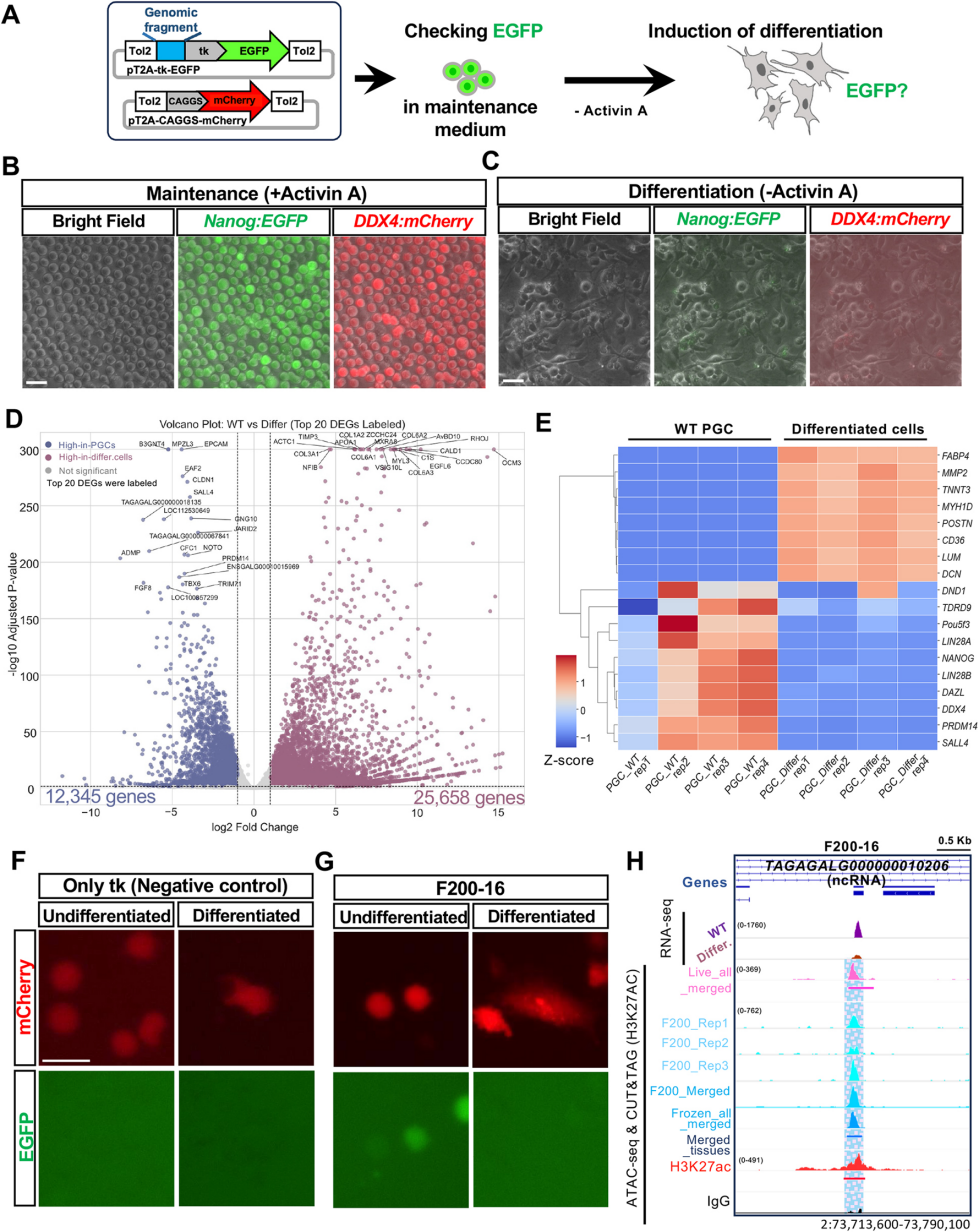
**A novel reporter assay system for monitoring PGC identity loss under Activin A-deficient conditions.** (A) Schematics of the novel *in vitro* PGC differentiation system. (B,C) Cultured chicken PGCs were co-transfected with a Nanog promoter-EGFP reporter (Nanog:EGFP) and a DDX4 promoter-mCherry reporter (DDX4:mCherry), and then either maintained in maintenance medium (+Activin A; B) or switched to differentiation medium (−Activin A; C). Under maintenance conditions, both Nanog:EGFP and DDX4:mCherry signals were readily detected, whereas under differentiation conditions both signals were abolished. Bright-field images are shown for reference. Scale bar: 30 µm. (D) Volcano plot showing comparative transcriptomic analysis between wild-type PGCs and differentiated cells. Differentially expressed genes (DEGs) significantly upregulated in wild-type PGCs are shown in slate blue, whereas those upregulated in differentiated cells are shown in mauve (q<0.05, ∣log2 FC∣>1). The top 20 DEGs are labeled with their respective gene symbols. (E) Heatmap illustrating the differential expression patterns of ten PGC markers and eight mesenchymal markers between wild-type PGCs and differentiated cells. Expression levels are normalized to Z-scores from −1 to 1 based on TPM. (F,G) Representative images of the enhancer activity assay. (F) Negative control construct and (G) enhancer construct [F200-16 (2: 73,751,403-73,752,394)]. EGFP expression observed under maintenance conditions was markedly reduced or lost upon differentiation, indicating PGC-specific enhancer activity. Scale bar: 25 µm. (H) IGV tracks displaying RNA-seq expression in wild-type PGCs (magenta) and differentiated cells (orange-red). The panel includes ATAC-seq signals from merged live PGCs (pink) and merged frozen PGCs (light blue), individual tracks for eight somatic tissues (blue), and CUT&Tag profiles for H3K27ac (red) with an IgG control (black) at a representative F200-16 genomic locus.

To assess whether the enhancer activity of PGC-specific ACRs depends on the undifferentiated state, we examined EGFP expression following *in vitro* differentiation of PGCs. During the course of this study, we found that cultured PGCs spontaneously differentiate when transferred to a medium lacking Activin A (Fig. 4A). Under these conditions, PGCs that typically remain in suspension adhered to the culture dish and displayed morphological features indicative of differentiation. Promoter activity of established germline genes such as *Nanog* and *DDX4* was markedly reduced in this condition, as evidenced by diminished reporter fluorescence (Fig. 4B,C).

To further define the transcriptional identity of cells following Activin A withdrawal, we performed RNA-seq analysis comparing differentiated cells (−Activin A) with cultured PGCs maintained under self-renewal conditions (+Activin A) (Fig. 4D,E, Fig. S6). Transcriptomic analysis revealed that 25,658 genes were upregulated in the differentiated cells (Fig. 4D, Table S10). Among these DEGs, there was significant downregulation of key PGC and stem cell markers, including *DDX4*, *DND1*, *DAZL*, *TDRD9*, *PRDM14*, *LIN28A*, *SALL4*, *Pou5f3* and *NANOG* in differentiated cells (Fig. 4E). In contrast, genes associated with mesenchymal lineage programs, including extracellular matrix and connective tissue components (*POSTN*, *DCN*, *LUM* and *MMP2*), adipogenic markers (*FABP4* and *CD36*), and contractile or muscle-associated genes (*TNNT3* and *MYH1D*). Together, these findings indicate that removal of Activin A leads to loss of germline identity and acquisition of a mesenchymal lineage-like transcriptional state (Fig. S6C). Although long-term culture systems for chicken PGCs are well established, defined conditions that reproducibly induce spontaneous differentiation have remained unclear. Our results establish a simple and reliable strategy to experimentally drive loss of germline identity *in vitro*.

Using this differentiation system, we cultured PGCs transfected with enhancer reporter constructs in the absence of Activin A, and monitored EGFP fluorescence before and after differentiation. Strikingly, EGFP signals driven by all enhancer candidates – including those with strong activity in undifferentiated PGCs, such as F200-4 (C-13), F200-5 (C-11), F200-8, F200-10, F200-16, C-1, C-3, C-4, C-12, C-15, C-16 and C-19 – were abolished following differentiation (Fig. 4F,G and Fig. S7), and their corresponding genomic regions exhibited consistent ATAC-seq peaks across the replicates of frozen 200-cell input samples (Fig. 4H and Fig. S4). In contrast, mCherry fluorescence from the co-transfected control plasmid remained detectable in both undifferentiated and differentiated cells (Fig. S7).

Together, these data support the interpretation that the tested ACRs represent germline-competent, state-dependent regulatory elements that are selectively active in undifferentiated PGCs. Notably, these enhancers were originally identified from ATAC-seq datasets generated even under low-input and cryopreserved conditions, highlighting the biological fidelity of the approach.

### PGC-specific enhancer activity is restricted to early development

**Fig. 5.**
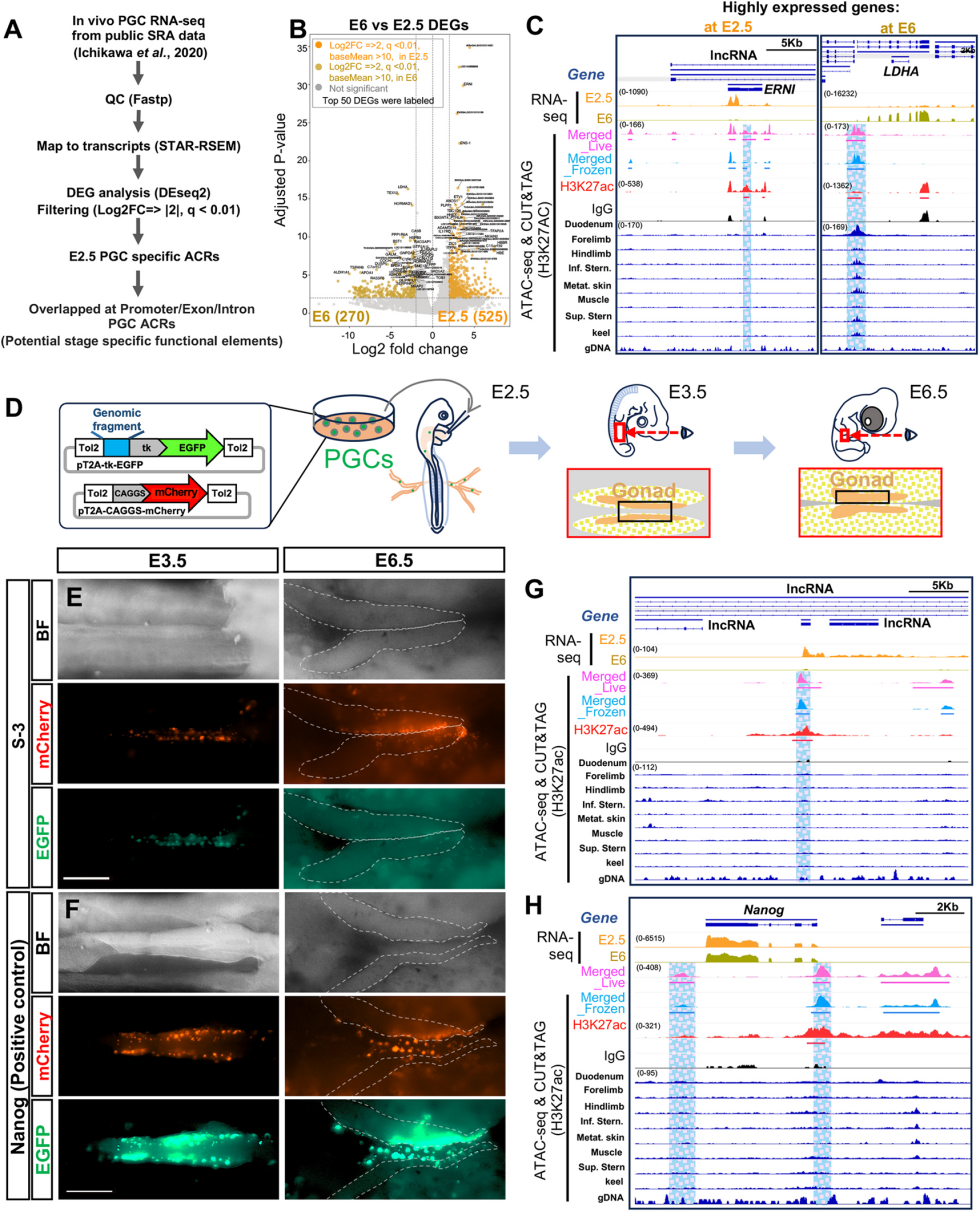
**PGC stage-specific ACRs exhibit enhancer activity *in vitro* and are downregulated upon colonization of the gonads.** (A) Schematic overview of the analysis workflow. (B) Volcano plots showing transcriptome re-analysis comparing *in vivo* PGCs at E2.5 and E6 (q<0.01, ∣log2 FC∣>2, baseMean>10). Genes highly expressed at E2.5 are marked with light orange; those at E6 are marked with orange. The top 50 most significantly expressed genes are labelled. (C) IGV tracks displaying RNA-seq expression in E2.5 (orange) and E6 (dark orange). The panel includes ATAC-seq signals from merged live PGCs (magenta) and merged frozen PGCs (light blue), individual tracks for eight somatic tissues (blue), and CUT&Tag profiles for H3K27ac (red) with an IgG control (black) at a representative F200-16 genomic locus. The left panel shows high expression of *ERNI* (known as early neural induction gene) at E2.5, while the right panel shows high expression of *LDHA* (known as lactate dehydrogenase A) at E6. (D) Schematic diagram of the transplantation assay. Cultured PGCs transfected with enhancer reporter constructs. CAGGS-mCherry and CAGGS-T2TP plasmids were injected into the bloodstream of E2.5 chicken embryos. Embryos were harvested at either E3.5 or E6.5. (E,F) Representative fluorescence images of transplanted PGCs at E3.5 and E6.5, shown as ventral views of the gonadal regions outlined in D. Bright-field, mCherry (transfection control) and EGFP (enhancer reporter) channels are shown. White dotted lines indicate the outline of the developing gonads. (E) S-3 enhancer construct. Robust EGFP fluorescence was detected at E3.5 but was absent at E6.5, whereas mCherry fluorescence persisted at both stages. (F) Nanog promoter-EGFP construct (positive control). Similar to the mCherry fluorescence pattern, robust EGFP fluorescence was observed throughout at the early to the late developmental stages. Scale bars: 500 μm. All experiments were performed with *n*=5 embryos per condition. (G,H) IGV tracks showing transcripts from E2.5 and E6, along with ATAC-seq signals from merged live PGCs and frozen PGCs, eight somatic tissues, H3K27ac and an IgG control (the same color coding as described in C). At the Nanog locus where Nanog expression is high at E2.5, strong ATAC-seq signals were observed around the TSS and/or TTS. Nanog transcripts were detectable at both E2.5 and E6.

Finally, we asked whether our ATAC-seq dataset could be leveraged to identify cis-regulatory elements that act in a stage-dependent manner during PGC development. We re-analyzed published RNA-seq data from endogenous chicken PGCs (Ichikawa et al., 2022) to define genes differentially expressed between migrating PGCs at embryonic day 2.5 (E2.5) and gonad-resident PGCs at E6 (DESeq2; baseMean>10, q<0.01, |log₂FC|>2), identifying 525 E2.5-upregulated and 270 E6-upregulated genes (Fig. 5A,B, Table S11). We then intersected these transcriptomic signatures with our PGC ATAC-seq profiles to extract ACRs located near E2.5-enriched genes, exemplified by *ERNI* (early neural induction gene) (Fig. 5C).

Among 10,550 filtered peaks, 291 PGC-specific ACRs were associated with E2.5-enriched genes, including 157 genes harboring at least one such ACR within 2.5 kb (Fig. 5C, Table S12). In contrast, loci of E6-enriched genes such as *LDHA* (lactate dehydrogenase A) showed primarily small, broadly shared accessibility and lacked PGC-specific ACRs (Fig. 5C). This pattern is consistent with the E2.5 origin of the cultured PGCs used for ATAC-seq, and suggests that the resulting accessibility landscape is particularly informative for identifying early-stage regulatory elements.

From the E2.5-linked candidate set, we designated a subset as putative stage-specific enhancers (S-series) and first evaluated them in cultured PGCs (Figs S7, S8 and S10; S-4 and S-5 are shown in Fig. S10). Candidates showing robust enhancer activity were further examined under differentiation conditions to confirm PGC-state dependence (Figs S3 and S9; Fig. S3 primarily contains F-series constructs, and S-3 corresponds to F200-16). Based on these *in vitro* screening steps, three candidates (S-3, S-4 and S-5) were selected for subsequent *in vivo* evaluation.

For transplantation, reporter-transfected PGCs were injected into the bloodstream of E2.5 embryos and analyzed at E3.5 (migratory/arrival phase) and E6.5 (gonad-resident phase) (Fig. 5D). All tested S-series enhancers drove EGFP expression at E3.5 (Fig. 5E,F; Fig. S10), but were largely silenced at E6.5, whereas mCherry from the co-transfected control plasmid remained detectable at both stages (Fig. 5E-H; Fig. S10). An exception was observed for S-4, in which strong EGFP fluorescence was detected at the midline in a subset of embryos at E6.5 (Fig. S10). These EGFP-positive cells were localized within the dorsal mesentery rather than inside the gonadal tissue, suggesting that they may represent PGCs that failed to complete migration to the gonadal niche.

By comparison, a *Nanog* promoter-driven reporter construct remained active at both E3.5 and E6.5 (Fig. 5F), consistent with sustained Nanog expression in gonad-resident PGCs (Choi et al., 2021). In contrast, the negative control construct showed minimal EGFP fluorescence at both developmental stages (Fig. S10).

To quantitatively compare stage-dependent enhancer activity, EGFP intensity was quantified and normalized to mCherry signal in cryosections (Fig. S11). For S-3, S-4 and S-5, EGFP intensity in migrating PGCs at E3.5 was significantly higher than in gonad-resident PGCs at E6.5. Notably, the strong EGFP-positive cells observed in S-4 at E6.5 corresponded to PGCs remaining within the mesentery, consistent with the whole-mount observation. Interestingly, Nanog promoter-driven EGFP expression increased at E6.5 compared to E3.5, in agreement with persistent Nanog transcription at later stages. A modest increase in background EGFP signal was observed in the negative control at E6.5 (Fig. S11); however, this trend was opposite to that seen in enhancer-containing constructs and did not affect the overall conclusion. Together, these results indicate that the tested ACRs function as enhancers that are active during early PGC migration but become selectively silenced following colonization of the gonadal niche.

In summary, we adapted Omni-ATAC-seq, which can generate reproducible and biologically informative chromatin accessibility profiles from as few as 200 cryopreserved chicken PGCs, allowing robust identification of functional cis-regulatory elements even under highly limited input conditions. While our current CUT&Tag analysis identified a relatively conservative number of H3K27ac-marked regions (673 peaks), it should be noted that ACRs encompass a broad spectrum of regulatory elements beyond active enhancers, including poised enhancers, active promoters and insulators characterized by other histone modifications such as H3K4me1 or H3K4me3. Therefore, while our H3K27ac profiling was not exhaustive, the rigorous integration of ATAC-seq and RNA-seq provided a high-confidence set of functional candidates, with a subset validated by our reporter assays. Future studies employing a more-comprehensive epigenetic landscape – incorporating additional histone marks and higher-order chromatin architecture – will be essential to fully resolve the multifaceted regulatory circuitry of chicken PGCs.

Furthermore, we established a novel *in vitro* culture system that reliably induces the spontaneous differentiation of chicken PGCs. By integrating the candidates of lineage- and stage-specific ACRs with functional reporter assays in this differentiation model and through *in vivo* transplantation, we demonstrate that meaningful, stage-dependent enhancer activity can be accurately extracted from even minimal cryopreserved material. Beyond its technical implications, our findings provide a framework to dissect the gene regulatory architecture underlying germline specification and developmental stage-specific transcriptional programs. Given the unique experimental accessibility of chicken PGCs, this approach will not only accelerate germline biology research but also enable comparative and functional studies across diverse developmental systems and rare cell populations.

## MATERIALS AND METHODS

### Animals, staging and animal care

Fertilized chicken (*Gallus gallus domesticus*, White leghorn) eggs were purchased from Yamagishi poultry farm (Mie, Japan) and from Nagoya University through the National Bio-Resource Project of the MEXT, Japan. Eggs were incubated at 38.5°C, and embryos were staged using Hamburger and Hamilton's stages (Hamburger and Hamilton, 1951). All animal experiments were performed with the approval of the Institutional Animal Care and Use Committees at Kyushu University under the approval number A25-189-1.

### Establishment of chicken cultured PGCs

Circulating PGCs, along with blood cells, were harvested from blood of HH15 female chicken embryos and were cultured on non-coated (non-adhesive) plates in calcium-free DMEM (Gibco) diluted with water, supplemented with Fibroblast Growth Factor Chimera (Wako), Activin A (APRO Science group) and chicken serum (Biowest) (FAcs medium), according to previously described methods (Chen et al., 2019; Whyte et al., 2015). After 1 month of expansion, PGCs were cryopreserved at −80°C in Bambanker (NIPPON Genetics) until further use in experiments.

### PGC frozen stock and ATAC-seq library preparation

ATAC-seq libraries from living and cryopreserved PGC were prepared based on the Omni-ATAC seq method with modifications, as recently published (Corces et al., 2017; Kawaguchi et al., 2024). Cultured PGCs were harvested, the cell density was counted, and the cells were aliquoted to the desired numbers (200, 1000, 5000 and 25,000) in individual DNA low binding tubes (Eppendolf, 0030108051). Cells were collected with centrifugation (350 ***g*** for 5 min at room temperature) (KUBOTA, 2410). For cryopreservation, collected cells were individually resuspended into 50 μl of Banbanker cryopreservation medium (Nihon Genetics, CS-02-001). Tubes were slowly frozen using Mr.Frosty (Thermofisher, 5100-0001) and kept at −80°C until library preparation. When ATAC-preparations were conducted, cryopreserved cells were thawed at 37°C for 5 min, and the cryopreserved samples were prepared together with living cultured PGC samples. Concurrently, living cultured PGCs were harvested as described above. In the cryopreserved and living cultured cells, 500 μl lysis buffer 1 was added [10 mM Tris-HCl (pH 7.5), 3 mM MgCl_2_, 2 mM NaCl and ×50 Protease inhibitor EDTA free (nacalai tesque, 03969)], and gently mixed and harvested (350 ***g*** for 5 min at 4°C) (KUBOTA, 2410). The cell pellets were resuspended in 50 μl lysis buffer 2 [10 mM Tris-HCl (pH 7.5), 3 mM MgCl_2_, 2 mM NaCl, 0.1% Tween-20, 0.1% NP-40, 0.01% digitonin and ×50 Protease inhibitor EDTA free (nacalai tesque, 03969)] and incubated on ice for 15 min. 450 µl of lysis buffer 3 [10 mM Tris-HCl (pH 7.5), 3 mM MgCl_2_, 2 mM NaCl and 0.1% Tween-20] was added to the cells and mixed gently. Immediately, cells were spun down (350 ***g*** for 5 min at 4°C) and pellets were resuspended with 25 μl of Tn5 Transposase reaction for an input of 200 and 1000 cells, and 50 μl Tn5 transposase reaction for an input of 5000 and 25,000 cells [Tn5 transposase reaction: 12.5 μl of the 2× transposase buffer (20 mM Tris-HCl at pH 7.5, 10 mM MgCl_2_ and 20% DMF), 16.5 μl of PBS, 0.5 μl of 10% Tween-20 (0.1% f. c.), 0.5 μl of 1% digitonin (0.01% f. c.) and 2.5 μl of oligo loaded in-house Tn5 transposase (0.5 μg of in-house Tn5 transposase stock was diluted in 1:10)]. Tagmentation proceeded at 37°C in a water bath for 1 h with occasional agitation. The reaction was stopped by adding five times the volume of PB (Qiagen, 19065), followed by vortexing for 30 s. Tn5 transposase treated DNA was purified with the MinElute PCR purification Kit (Qiagen, 28004) and eluted with 20 μl of EB buffer. 10 μl of purified DNA solution was used for library amplification, and the final amplification cycles were defined with intermediate qPCR, as described in the original ATAC-seq method (Buenrostro et al., 2013). Nonetheless, all libraries were amplified with 9-16 total PCR cycles. Final libraries were amplified with NEBNext High-Fidelity 2XPCR Master Mix (NEB, M0541) with Nextera Unique Dual index (UDI) adapter plate A (Illumina, 1000000002694). After PCR amplification, the libraries were purified with magnetic beads for DNA isolation. Removing Adapters were prepared according to the manufacturer's instructions. Sequencing was performed using Nova-seq6000 PE150 (paired end 150 bp) by a third party (Novogene).

### CUT&Tag library preparation

To validate histone marks, CUT&Tag libraries were prepared using the following protocols (Kawaguchi et al., 2024; Kaya-Okur et al., 2019; dx.doi.org/10.17504/protocols.io.wnufdew), with minor modifications, as described below. Library preparation was initiated with 10,000 cultured PGCs. Harvested cells were pelleted by centrifugation (350 ***g*** for 5 min at room temperature). The cell pellet was resuspended in nuclei extraction (NE) buffer [20 mM HEPES-KOH (pH 7.9), 10 mM KCl, 0.1% Triton X-100, 20% glycerol and 0.5 mM spermidine] supplemented with EDTA-free protease inhibitor (nacalai tesque, 03969) (hereafter, NE+PI) and incubated for 10 min with occasional agitation. Nuclei were collected by centrifugation (350 ***g*** for 5 min at room temperature), and the pellets were resuspended in 100 μl cold NE+PI per 2×10⁴ cells. The nuclei suspension was transferred to a PCR tube and mixed with 11 μl Concanavalin A-Conjugated Paramagnetic Beads (Epicypher, 21-1411) per 20,000 cells, pre-washed in NE buffer and activated according to the manufacturer's protocol. The nuclei-bead slurry was incubated for 10 min at room temperature. Tubes were placed on a magnetic stand until the slurry cleared, and the supernatant was removed. The bead-bound nuclei were washed twice with 50 μl cold AB150 [20 mM HEPES-KOH (pH 7.9), 150 mM NaCl, 0.5 mM spermidine, 0.01% digitonin, 2 mM EDTA, Roche cOmplete, Mini, EDTA-free protease inhibitor (nacalai tesque, 03969)] with gentle pipetting, then resuspended in 50 μl cold AB150. Primary antibody was added and incubated overnight at 4°C: 0.75 μl H3K27ac (Diagenode, C15200184; 1:1000) or 0.5 μl normal rabbit IgG (Cell Signaling Technology, 2729; 1:1000) for control. After incubation, tubes were placed on the magnet and washed twice with 100 μl DIG150 [20 mM HEPES-KOH (pH 7.9), 150 mM NaCl, 0.5 mM spermidine, 0.01% digitonin and EDTA-free protease inhibitor (nacalai tesque, 03969)]. Beads were resuspended in 50 μl ice-cold DIG150 and 0.75 μl secondary antibody (Novus Biologicals, NBP1-72763 for anti-mouse; 1:1000) was added and gently mixed. Tubes were incubated for 30 min at room temperature. Beads were placed on the magnet, washed twice with ice-cold DIG300 [20 mM HEPES-KOH (pH 7.9), 300 mM NaCl, 0.5 mM spermidine, 0.01% digitonin and EDTA-free protease inhibitor (nacalai tesque, 03969)], and resuspended in 50 μl DIG300. pAG-Tn5 (EpiCypher, SKU 15-1017) was added to a final amount of 2.5 μl, mixed gently and incubated on a shaker for 1 h at room temperature. Tubes were returned to the magnet and washed twice with 100 μl DIG300. Beads were resuspended in 50 μl cold tagmentation buffer (DIG300 and additional 10 mM MgCl₂) and incubated at 37°C for 1 h with occasional gentle agitation. After tagmentation, 50 μl of 0.4% SDS (final concentration; 0.2%) was added and incubated for 10 min at room temperature. For DNA release, 1 μl Proteinase K was added and samples were incubated for 1 h at 50°C. DNA was purified using 1.8× AMPure beads, following the manufacturer's protocol. Libraries were then amplified with NEBNext High-Fidelity 2X PCR Master Mix (NEB, M0541): 13 cycles for H3K27ac libraries and 15 cycles for IgG libraries. Final libraries were purified using magnetic beads. Sequencing was performed in paired-end 150 bp (PE150) mode.

### Computational analysis and data visualization

#### Reference genome and annotation versions

Throughout the analysis, the reference genome assembly bGalGal1.mat.broiler.GRCg7b (Accession Number GCF_016699485.2) and gene annotation from Gallus Enriched Gene Annotation were used as reference gene models (Degalez et al., 2024) (https://gega.sigenae.org).

### ATAC-seq and CUT&Tag data processing and peak analysis

#### Data processing from raw sequencing reads to BigWig generation for visualization

For both ATAC-seq and CUT&Tag data, trimming of Nextera adapters and filtering of low-quality reads were performed using Fastp (v0.23.2) with the parameters (-q 15 -n 10 -t 1 -T 1 -l 20) (Chen et al., 2018). Bowtie2 (v2.5.4) (Langmead et al., 2019) was used for read mapping with the parameters (--no-mixed --no-discordant -X 1000). SAMtools (v1.17) (Danecek et al., 2021) was used to convert SAM files to BAM files, and sorting and indexing were performed with default settings. Picard (v3.0.0) (https://broadinstitute.github.io/picard/), was used for duplicate removal and fragment size distribution analysis with the MarkDuplicates and CollectInsertSizeMetrics tools. Duplicate-removed BAM files were further used to assess mtDNA and unmapped reads using SAMtools with the parameters (view -f 4). Peaks were detected with MACS3 peakcall (v3.0.0a6), and used the --broad option to account for the nature of accessible regions spanning multiple nucleosomes with the parameters (-f BAMPE -q 0.05 -g 1053332251 --broad) (Zhang et al., 2008). Peak comparisons between H3K27ac and IgG were performed using Bedtools (v2.31.0) (Quinlan and Hall, 2010), and annotations were carried out using annotatePeaks.pl from HOMER (v4.9.1) (Heinz et al. 2010; http://homer.ucsd.edu/homer/).

##### For ATAC-seq peak processing

To identify potential PGC-specific ACRs, we focused on ACRs detected in both live and cryopreserved samples. For binary comparisons, peaks across all samples were intersected using Bedtools (v2.31.0). To exclude gDNA-derived peaks from the PGC ATAC-seq data, overlapping peaks were identified with Bedtools intersect with the parameters (-u -f 0.5) and subsequently subtracted. Peaks were ranked based on p-values and q-values obtained from MACS3. Peak comparisons were performed using Bedtools (v2.31.0) (Quinlan and Hall, 2010), and annotations were carried out using annotatePeaks.pl from HOMER (v4.9.1) To validate enhancer activity *in vivo* and *in vitro*, peaks were filtered and selected based on MACS3 fold-change scores. For the C-series, the top 19 peaks with the highest fold changes were chosen. Of these, 12/19 peaks were successfully cloned into vectors and tested in our systems. In total, 12 constructs were validated *in vitro.* For the F-200 series, the peaks that were identified under the low-input condition (frozen 200 cells), we first chose peaks commonly detected across triplicates (15,218 peaks filtered bedtools multiinter with a threshold of N>3). After removing peaks shared with eight somatic tissues, 1284 peaks were remained. We then defined the meanFold changes across consensus regions of those peaks using bedtools map (option -o mean). Then, the top meanFold score of 16 peaks were selected. In these 16 peaks, four ACRs were overlapped with C-series. Eventually, 12 peaks were successfully cloned into vectors and tested in our systems; we named these F-200 series constructs. Ten constructs were validated *in vitro.* The UpSet plot was generated by intervene (v0.6.5) (Khan and Mathelier, 2017; Quinlan and Hall, 2010). For motif analysis, 1023 peaks from gDNA tagmentation and 1023 ACRs from merged live PGC samples (randomly selected using Bedtools) were converted to FASTA format and analyzed for enriched motifs using MEME (Bailey et al., 2015).

### RNA-seq analysis from published datasets

RNA-seq datasets were obtained from the publicly available data of Ichikawa et al. (2022), consisting of four biological replicates each of *in vivo* PGCs at E2.5 and E6. The datasets were obtained from the Gene Expression Omnibus under accession number GSE188689. Trimming of Nextera adapters and filtering of low-quality reads were performed using Fastp (v0.23.2) with the parameters (-q 15 -n 10 -t 1 -T 1 -l 20 -w 16) (Chen et al., 2018). Mapping and generation of count matrices were conducted using the STAR-RSEM pipeline with default parameter (Dobin et al., 2013; Li and Dewey, 2011). Differential expression analysis was carried out using PyDESeq2 (cutoff: baseMean >10, *q*>0.01) (Muzellec et al., 2023). For visualization, fastq files processed by Fastp were mapped using STAR to generate “Aligned.toTranscriptome.bam” files. Final bigWig files were generated using deepTools (v3.1.2) with the parameters (--binSize 10 --normalizeUsing RPKM --smoothLength 30 --effective GenomeSize 1053332251).

### RNA-seq preparation and analysis from *in vitro* differentiation samples

For the transcriptomic analysis of *in vitro*-differentiated cells, RNA-seq libraries were prepared using a modified SMART-seq2 protocol (https://dx.doi.org/10.17504/protocols.io.pbgdijw) (Picelli et al., 2014b; Trombetta et al., 2014). Total RNA was isolated from wild-type and differentiated PGCs using Trizol reagent (Thermo Fisher Scientific, 15596026). RNA concentration was measured using a Qubit fluorometer. For reverse transcription and subsequent cDNA amplification, 1 ng of total RNA was used to perform 16 cycles of reverse transcription and 16 cycles of PCR, respectively. The resulting cDNA was purified using 2× volumes of Ampure XP beads (Beckman Coulter) and eluted in 10 μl of nuclease-free water. For library preparation, 100 ng of amplified cDNA was subjected to tagmentation using Tn5 transposase at 55°C for 7 min. Tagmented cDNA was purified with Ampure XP beads, and sequencing libraries were generated using Illumina Unique Dual Indexes (UDI) with six cycles of PCR amplification. To remove adapter dimers and high molecular weight fragments, library size selection was performed using Ampure XP beads according to the manufacturer's protocol. The final libraries were sequenced on the NovaSeq X Plus platform (Illumina) by a third-party service provider. After obtaining the raw sequencing data, RNA-seq analysis was performed following the same pipeline used for the published datasets. Briefly, adapter trimming and quality filtering were conducted using fastp (v0.23.2) with the parameters (-q 15 -n 10 -t 1 -T 1 -l 20 -w 16) (Chen et al., 2018). Read mapping and quantification were performed using the STAR-RSEM (v1.3.3) pipeline with the --star option, employing STAR (v2.7.10) to generate count matrices (Dobin et al., 2013; Li and Dewey, 2011). Differential expression analysis was carried out using PyDESeq2 (Muzellec et al., 2023) with a threshold of baseMean>10 and an adjusted *P<*0.05. For visualization, bigWig files were generated from the genome-mapped BAM files using deepTools (v3.1.2) bamCoverage with the parameters (--binSize 10 --normalizeUsing RPKM --smoothLength 30 -- effective GenomeSize 1053332251). Gene Ontology (GO) analysis was performed using g:Profiler (https://biit.cs.ut.ee/gprofiler/gost) (Kolberg et al., 2023) and all data visualizations were implemented in R.

### Tn5 transposase purification and assembly

The pTXB1-Tn5 transposase expression plasmid (Addgene #60240) was digested with XbaI and BamHI, and a Tn5 transposase-intein-CBD fragment was subcloned into the pE21a (+) vector (NovoPro, V011023) as described by Sato et al. (2019). Tn5 transposase was expressed and purified as described previously (Picelli et al., 2014a; Sato et al., 2019), with the following modification. The *Escherichia coli* Rosetta 2 cells (DE3) carrying the Tn5 transposase expression plasmid were grown in 3 l (1.5 l×2) of Luria-Broth (LB) medium containing 100 µg/ml ampicillin and 34 µg/ml chloramphenicol at 37°C until the optical density reached 0.9 at 600 nm. The cell cultures were cooled to 10°C and further cultivated for 15 h after addition of isopropyl β-D-1-thiogalactopyranoside (IPTG) to a final concentration of 0.25 mM to induce protein expression. Following a further 4 h cultivation at 23°C, the cells were harvested by centrifugation and resuspended in 50 ml of lysis buffer [20 mM HEPES-KOH (pH 7.2), 0.8 M NaCl, 1 mM EDTA, 0.2% (w/v) Triton X-100 and 10% (w/v) glycerol] supplemented with cOmplete protease inhibitor cocktail (EDTA-free, Merck). The cells were disrupted by sonication and clarified by centrifugation at 28,000 ***g*** at 4°C for 45 min. 50 ml of the cell lysate (supernatant fraction) was gradually mixed with 5 ml of 10% (v/v) polyethyleneimine (pH 7.5), after which the mixture was incubated at 4°C for 20 min. It was then clarified by centrifugation at 20,000 ***g*** at 4°C for 20 min. The resulting supernatant fraction was mixed with 85 ml of lysis buffer containing protease inhibitor and then applied to a 17 ml bed volume of chitin resin (New England BioLabs). The resin was then washed with 250 ml of lysis buffer followed by 20 ml of lysis buffer containing protease inhibitor and 100 mM dithiothreitol (DTT). The resin was resuspended in the same buffer and incubated at 4°C for 36 h. The eluate was dialyzed against D1 buffer [25 mM HEPES-KOH (pH 7.2), 0.2 M NaCl, 0.2 mM EDTA, 0.2% (w/v) Triton X-100, 20% (w/v) glycerol] for 15 h. The dialyzed fraction was applied to a 1 ml Resource S column (Cytiva). The column was developed with a linear gradient of 0.2 to 1 M NaCl in H buffer [20 mM HEPES-KOH (pH 7.2), 0.2 mM EDTA, 20% (w/v) glycerol] totaling 15 ml. The peak fractions were collected and dialyzed against D2 buffer [100 mM HEPES-KOH (pH 7.2), 0.2 M NaCl, 0.2 mM EDTA, 10% (w/v) glycerol] at 4°C for 15 h and then mixed with glycerol equivalent to 0.8 volume of the dialyzed fraction. The aliquots were snap-frozen in liquid nitrogen and stored at −80°C. Tn5 transposase loading with oligo was produced as described by Picelli et al. (2014a). Tn5 transposase loaded with Oligo was kept at −20°C for up to 1 month.

### Plasmid constructions

#### pT2A-tk-EGFP

The pT2AL200R150G vector (Kawakami, 2005) was digested with XhoI and BglII. A DNA fragment containing multiple cloning sites (MCS), the tk promoter, EGFP coding sequence, and polyadenylation (polyA) sequence was amplified by PCR from ptkEGFP (Kawakami, 2005; Uchikawa et al., 2003). This fragment was then recombined with the digested the pT2AL200R150G vector using the In-Fusion HD cloning kit (TAKARA).

#### pT2A-CAGGS-mCherry

The pT2A-CAGGS-MCS1-2A-MCS2 vector (Saito et al., 2022) was digested with NotI and MluI. A mCherry coding sequence was amplified by PCR. This fragment was ligated into the digested the pT2A-CAGGS-MCS1-2A-MCS2 by Mighty Mix DNA Ligation Kit (TAKARA).

#### pT2A-Genomic fragment:tkEGFP

The genomic region of interest was isolated and amplified by PCR from the genome of BL-E strain chickens maintained at Nagoya University. The amplified fragments were digested with XhoI-EcoRV, XhoI or EcoRV-BglII and subsequently ligated into XhoI-EcoRV- or EcoRV-BglII-digested pT2A-tkEGFP.

#### pT2A-Nanog:EGFP

The pT2AL200R150G vector was digested with XhoI and BglII. A DNA fragment containing multiple cloning sites (MCSs), an EGFP-coding sequence and polyadenylation (polyA) sequence was recombined with the digested the pT2AL200R150G vector using the In-Fusion HD cloning kit (Takara), resulting in the pT2A-MCS-EGFP plasmid. A 4000 bp upstream region from the Nanog start codon was isolated and amplified by genomic PCR from BL-E strain chickens. This fragment was ligated into the MluI-EcoRV site of pT2A-MCS-EGFP.

#### pT2A-MVH(DDX4):mCherry

The pT2AL200R150G vector was digested with XhoI and BglII. A DNA fragment containing MCSs, the mCherry coding sequence and the polyadenylation (polyA) sequence was recombined with the digested the pT2AL200R150G vector using the In-Fusion HD cloning kit (Takara), resulting in the pT2A-MCS-mCherry plasmid. An ∼2000 bp upstream genomic region from the start codon of the mouse VASA homolog (MVH), kindly provided from Dr Bertrand Pain (Stem Cell and Brain Research Institute, Bron, France and Université de Lyon, France), was amplified and recombined with the MluI-EcoRV-digested pT2A-MCS-mCherry vector using the In-Fusion HD cloning kit. KOD One (Toyobo) was used for all PCR reactions. Primers and amplified genomic fragments are listed in Table S5.

### Plasmid transfection

A total of 5×10^4^ cultured PGCs were washed with OPTI-MEM (Gibco) and placed into a 96-well coated plate containing 100 μl of FAot medium (adding Ovo-transferrin instead of chicken serum) (Whyte et al., 2015) without heparin and antibiotics (penicillin, streptomycin and amphotericin) for 3 h. A transfection mixture containing 0.2 μg of total plasmids and 0.5 μl of Lipofectamine 2000 (ThermoFisher Scientific) in 50 μl of OPTI-MEM was then added to the wells. The ratio of plasmid concentrations used was 3:1 for reporter vector : CAGGS-mCherry vector and an appropriate amount of Tol2 transposase vector (Sato et al., 2007) was co-transfected accordingly. 6 h after transfection, the medium was replaced with conventional FAcs medium (maintenance medium) containing heparin and antibiotics.

### Induction of PGC differentiation

After transfection, PGCs were cultured for 1 week in a maintenance medium. Subsequently, the cells were transferred to tissue culture-treated plates containing differentiation medium, which was prepared by omitting Activin A from the standard maintenance medium. The medium was refreshed by replacing half of the volume with fresh differentiation medium every 2 days. In the experiment, male PGCs were used, followed by transcriptomic analysis.

### PGC transplantation

Transfected PGCs were resuspended in Opti-MEM (Gibco) at a concentration of 5000 cells/µl and injected into the bloodstream of host chicken embryos (HH15-16) through the heart or dorsal aorta using a fine glass capillary. Following injection, embryos were incubated at 38.5°C and collected after 1 or 4 days of development, corresponding to E3.5 and E6.5, respectively.

### Cryosection preparation, immunostaining and quantification

Immunostaining of chicken embryo sections was performed as previously described (Atsuta et al., 2022). Embryos were fixed in 4% PFA in PBS for 3 h at 4°C, cryoprotected in a sucrose/PBS gradient (10% to 30%) and embedded in OCT compound (Sakura Finetek). Cryosections (10 μm) were prepared and dried overnight at room temperature. The sections were washed with 0.5% Triton X-100/PBS for 10 min at room temperature, incubated with 10% BSA blocking buffer (Thermo Fisher Scientific, J61655AK) for 30 min at room temperature and then incubated with primary antibodies overnight at 4°C. After washing twice with 0.5% Triton X-100/PBS, sections were incubated with secondary antibodies and DAPI (1:5000). Following secondary antibody incubation, sections were washed three times for 5 min each with 0.5% Triton X-100/PBS and imaged using a Zeiss LSM 900 confocal microscope. For immunohistochemical staining, the following antibodies were used: mouse anti-GFP (1:1000; MBL, M048-3), anti-RFP pAB (1:1000; MBL, PM005), Alexa Fluor 488 anti-mouse IgG (Thermo Fisher Scientific, A-11001) and Alexa Fluor 546 anti-rabbit IgG (Thermo Fisher Scientific, A-11035).

To measure GFP and mCherry intensities, laser power settings were calibrated using control sections transfected with the tk-control sections. Using the “Measure” function in ZEN Black software, the 488 nm laser power was minimized such that the intensity of mCherry-positive cells remained below 1.0 (to minimize bleed-through). The 546 nm laser intensity was adjusted to the maximum level without reaching saturation, as monitored by the Range Indicator. All images were acquired using these identical settings.

### Imaging

Fluorescence images of cultured cells were acquired using an inverted microscope (IX83, Olympus) equipped with an sCMOS camera (Zyla-4.2 plus, ANDOR) and operated with cellSens software (Olympus). Embryo images were captured using a cooled CCD camera (ORCA-R2, HAMAMATSU Photonics) mounted on a macro zoom microscope (MVX10, Olympus) and controlled by High Speed Recording (HSR) software (HAMAMATSU Photonics).

### Use of artificial intelligence tools

During manuscript editing, Google Gemini and DeepL Write were used for text style improvement.

## Supplementary Material



10.1242/develop.205214_sup1 Supplementary information

Table S1. Details of ATAC-seq libraries prepared in this study

Table S2. SRA data for ATAC-seq details and accession numbers utilized in the analysis

Table S3. Annotated peak features using HOMER annotatePeaks.pl Supplementary

Table S4. Genomic location of PGC-specific ACRs

Table S4. Genomic location of PGC-specific ACRs

Table S6. The list of Gene Ontology (GO) results using the gene sets from

Table S7. ACR information validated *in vivo* and *in vitro*

Table S8. Genomic location of F-200 series of enhancers and their meanFold changes between F200 replicates

Table S9. Peak information of peaks from H3K27ac signals

Table S10. Differentially expressed genes in WT_PGC vs Differ_cells by *in vitro* differentiation systems

Table S11. Differentially expressed genes in E2.5 vs E6 in vivo PGCs Supplementary

Table S12. Identification of PGC-specific ACRs associated with E2-high genes
