# Supplementary material for: ATAC-seq of low-input and cryopreserved primordial germ cells reveals functional enhancers
Source: Development. 2026 May 12;153(9):dev205214. doi: 10.1242/dev.205214 (PMC13245901; doi:10.1242/dev.205214)
Supplement: Supplementary information [file develop-153-205214-s1.pdf]

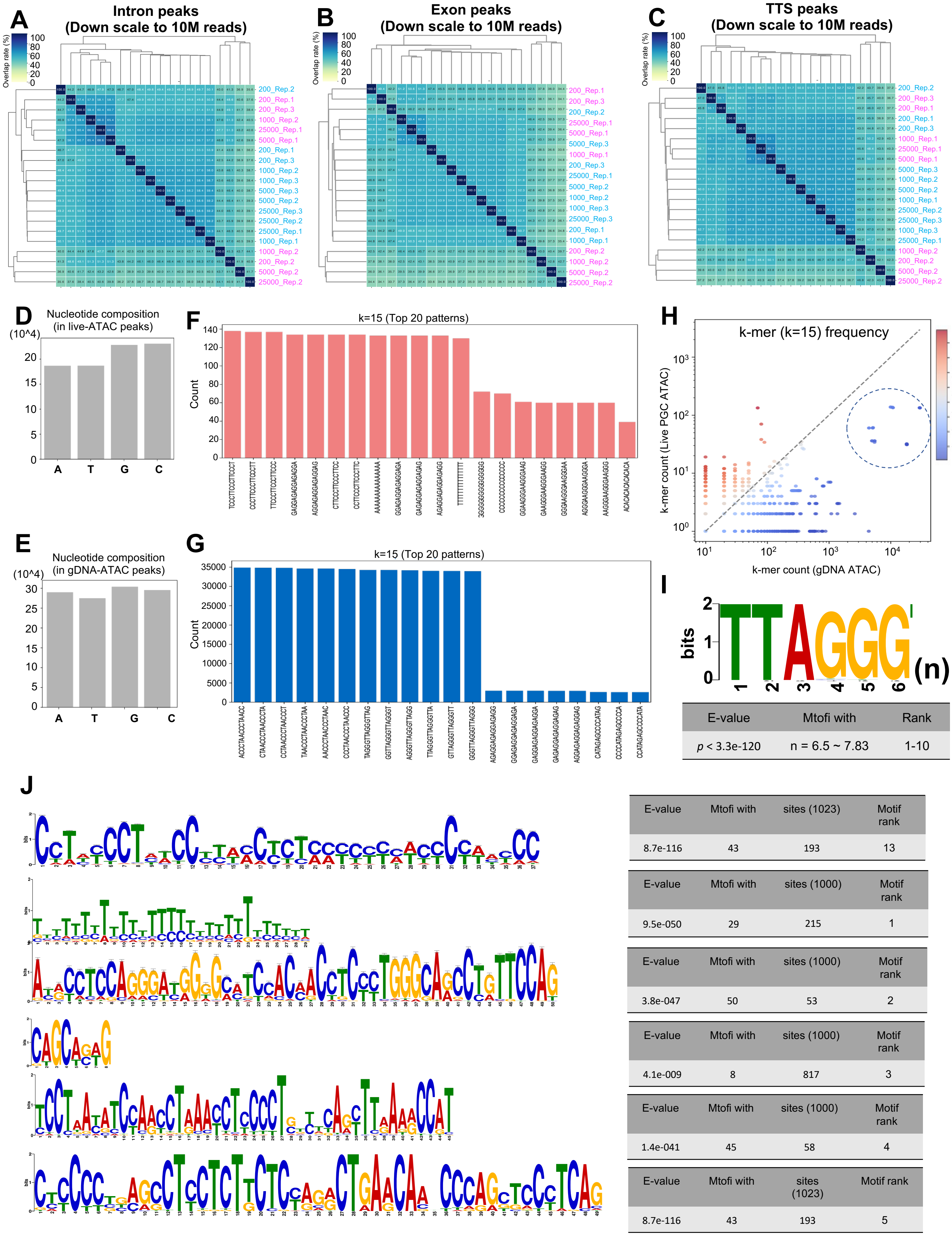

Development • Supplementary information

Fig. S1. Evaluation of peak overlap, sequence bias, and motif enrichment in control and experimental samples.

(A-C) Heatmaps showing the percentage of peak overlap across samples for intronic and exonic regions, and transcription termination sites (TTS) based on HOMER annotations. These features exhibited lower reproducibility (~60–65%) compared to promoter peaks, regardless of input condition. (D-E) Nucleotide composition analysis of live-cell peaks and gDNA control peaks. For the comparison, 1,023 peaks were randomly sampled from the live-cell dataset to match the number of peaks identified in the gDNA library. The live-cell peaks show a mild GC bias, likely reflecting promoter enrichment. In contrast, gDNA peaks displayed no strong nucleotide bias. (F-G) K-mer analysis (k=15) revealed strong sequence preference in the gDNA peaks, indicative of Tn5 transposase sequence bias, which was not observed in live-cell peaks. (H) Frequency distribution of control-biased sequences found in gDNA peaks, confirming that gDNA libraries tend to capture non-specific, sequence-driven Tn5 transposase activity. (I) MEME motif analysis of gDNA-derived peaks revealed highly enriched tandem repeats of the canonical telomeric sequence (TTAGGG)n, with 6.5 - 7.8 repeats per motif. These sequences were frequently located in intergenic regions and likely correspond to interstitial telomeric sequences (ITSs), emphasizing that peaks from gDNA reflect intrinsic sequence preferences rather than chromatin accessibility. (J) In contrast, MEME analysis of live-cell ATAC-seq peaks show no significant enrichment for repetitive telomeric motifs, further supporting that accessible chromatin profiles in live samples reflect true biological regulation. Motifs in panels are ranked by their MEME assigned E-value.

Development • Supplementary information

A

Chr.	Peaks PGC&Tissue	Peaks PGC_specific	Chr. Size (bp)
1	7969	1598	196449156
2	6339	1266	149539284
3	5222	1150	110642502
4	4624	993	90861225
5	3382	631	59506338
6	2294	492	36220557
7	2104	451	36382834
8	1856	409	29578256
9	1670	315	23733309
10	1440	288	20453248
11	1204	220	19638187
12	1506	323	20119077
13	1428	306	17905061
14	1168	257	15331188
15	1038	221	12703657
16	343	50	2706039
17	1039	251	11092391
18	956	199	11623896
19	867	178	10455293
20	1172	271	14265659
21	560	119	6970754

Chr.	Peaks PGC&Tissue	Peaks PGC_specific	Chr. Size (bp)
22	348	76	4686657
23	575	113	6253421
24	587	167	6478339
25	359	49	3067737
26	526	104	5349051
27	584	119	5228753
28	581	122	5437364
29	70	15	726478
30	86	10	755666
31	144	38	2457334
32	17	4	125424
33	229	39	3839931
34	391	117	3469343
35	54	1	554126
36	35	2	358375
37	24	8	157853
38	87	14	667312
39	26	2	177356
W	267	122	9109940
Z	2405	464	86044486

B

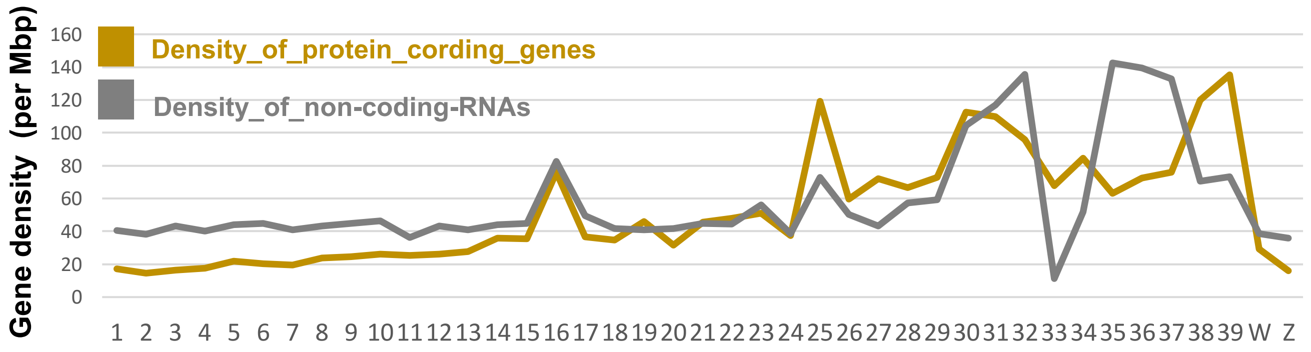

$$\text{Density} = \frac{\text{The number of feature}}{\text{The size of chromosome}} \text{ x M base}$$

Fig. S2. Chromosome-wide distribution of common and PGC-specific ACRs. (A) The table shows that per-chromosome counts of peaks shared between PGCs and tissues, and peaks classified as PGC-specific, together with chromosome sizes. (B) Densities of annotated protein-coding genes and non-coding RNAs per chromosome (features per Mbp).

Development • Supplementary information

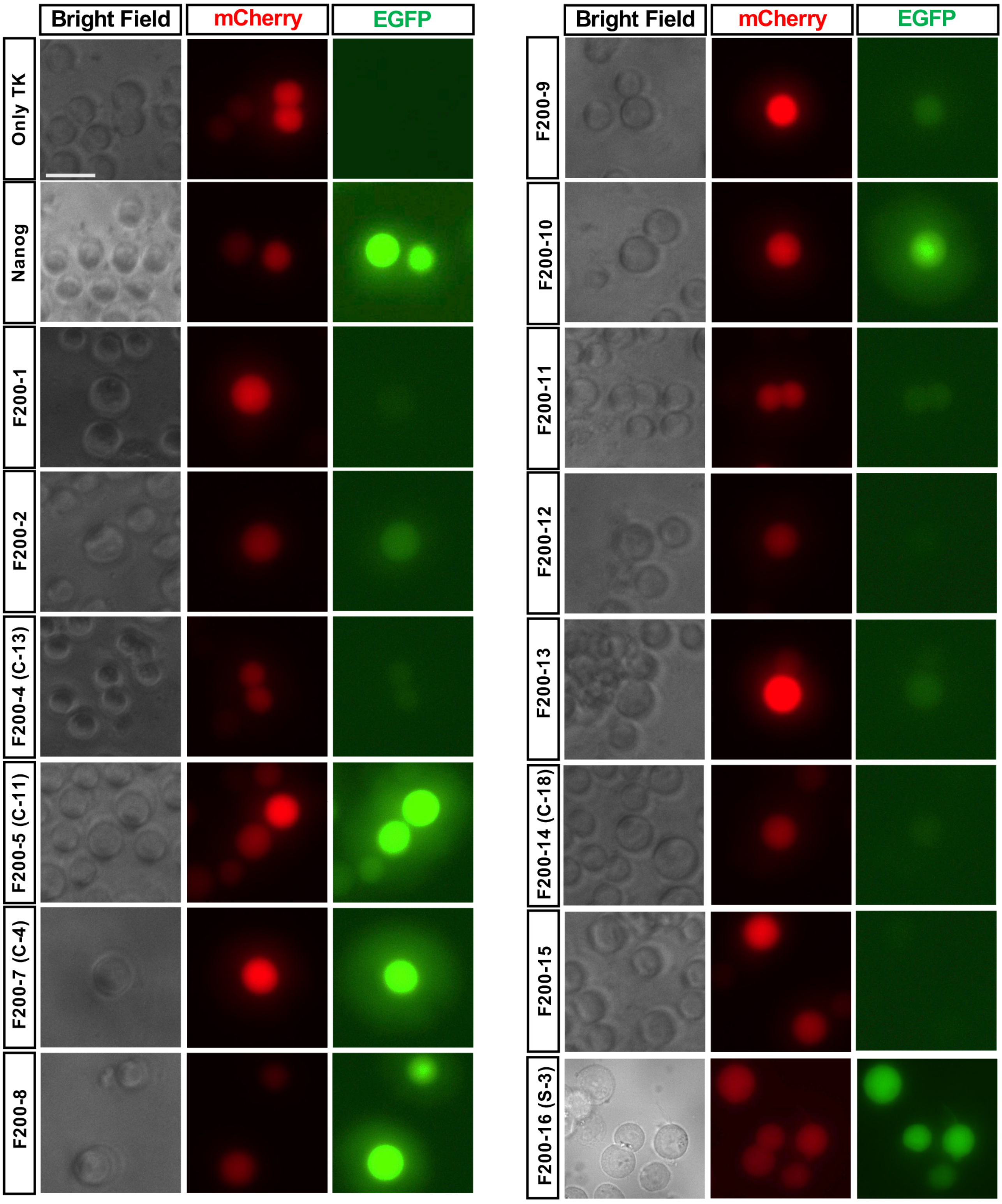

Development • Supplementary information

Fig. S3. *In vitro* screening of F-200 series candidate enhancer fragments in cultured PGCs.

Representative fluorescence images of cultured PGCs transfected with individual Tol2-based enhancer-EGFP reporter constructs. Genomic fragments (primarily F200-series candidates; selected overlapping C-series and S-series fragments, representing the same ACRs, are indicated in the labels) were cloned upstream of a minimal tk promoter driving EGFP. PGCs were co-transfected with enhancer–EGFP reporters, CAGGS-mCherry (transfection control), and CAGGS-T2TP plasmids. Images were acquired under maintenance conditions (+Activin A). For each construct, bright-field, mCherry, and EGFP channels are shown. The tk-EGFP vector lacking an upstream enhancer (Only tk) served as a negative control, and a Nanog promoter-EGFP construct was included as a positive control. Scale bars: 25 μm. Images are representative of three independent transfection experiments.

Development • Supplementary information

A

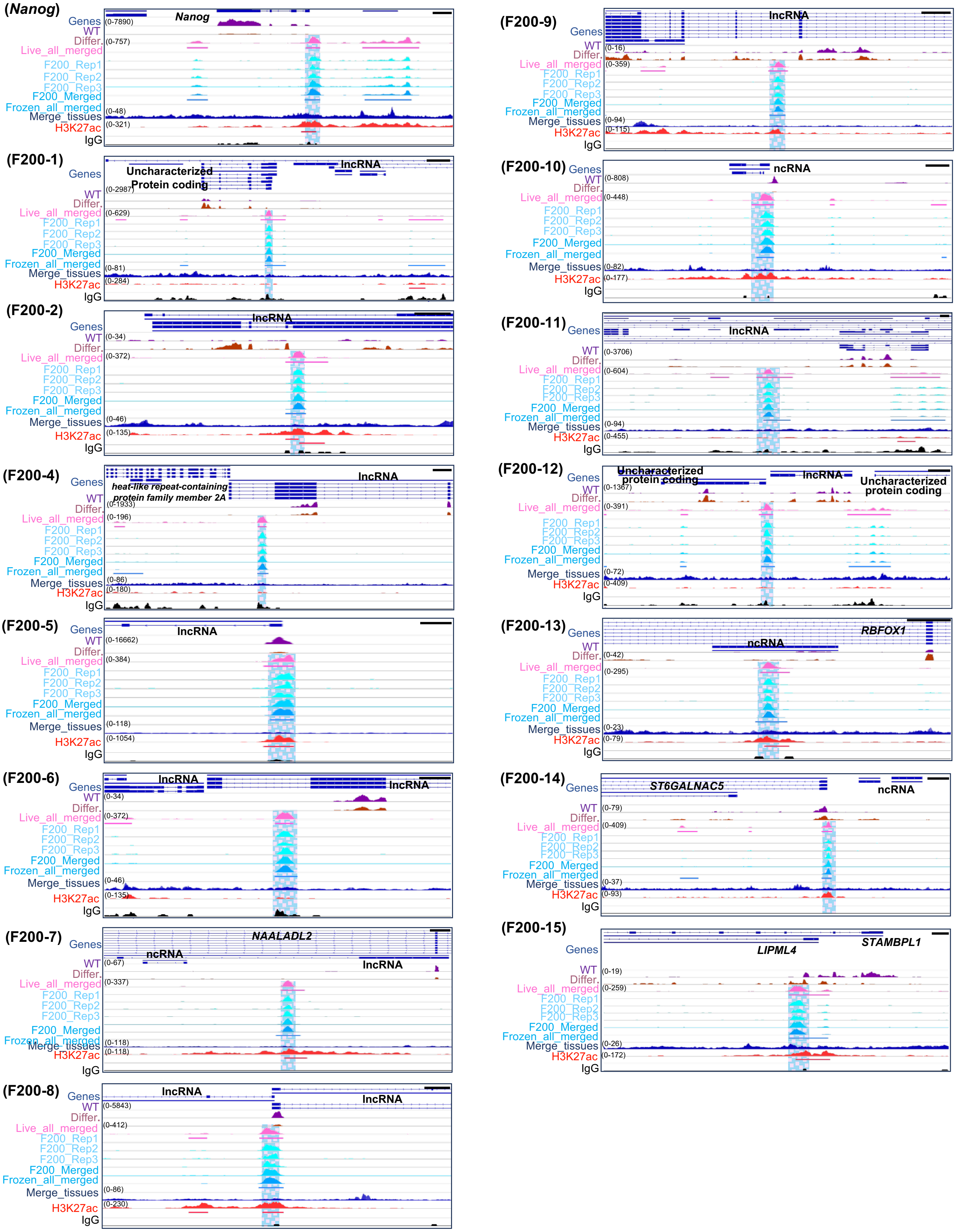

Development • Supplementary information

Fig. S4. Genome tracks of F200-enhancer candidates which were validated by *in vitro* differentiation analysis.

IGV tracks of F200-series loci identified from 200-cell frozen ATAC-seq peaks. Tracks showing as follows (top to bottom): gene models, RNA-seq (WT and differentiated PGCs), ATAC-seq (merged live PGCs, three biological replicates of 200-cell frozen PGCs, merged frozen PGCs, and merged somatic tissues), and H3K27ac CUT&Tag. Scale bars represent 1 kb. Transcripts corresponding to long non-coding RNAs are labelled as "uncharacterized lncRNA" above the respective tracks. Genomic coordinates for each peak and their nearest promoters are listed in Supplementary Table 7.

Development • Supplementary information

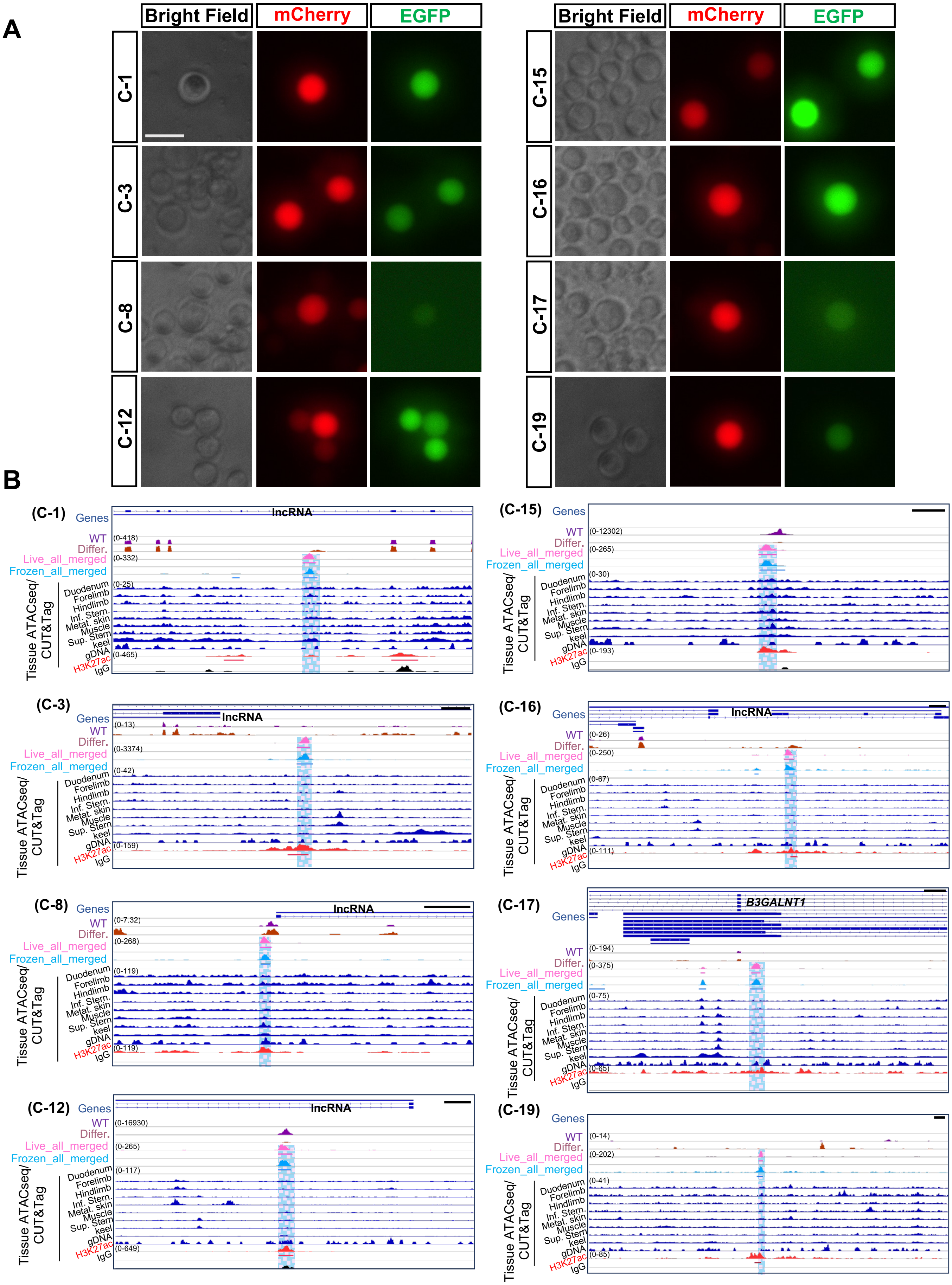

Development • Supplementary information

Fig. S5. ***In vitro*** **screening of M-series candidate enhancer fragments in cultured PGCs and the corresponding genome tracks.**

Additional M-series candidate fragments not included in Supplementary Figure 2 were screened using the same *in vitro* reporter assay. Representative fluorescence images of cultured PGCs transfected with individual M-series genomic fragments cloned upstream of a minimal tk promoter driving EGFP. PGCs were co-transfected with enhancer–EGFP reporters, CAGGS-mCherry (transfection control), and CAGGS-T2TP plasmids. Images were acquired under maintenance conditions (+Activin A). For each construct, bright-field, mCherry, and EGFP channels are shown. Scale bars: 25 µm. Images are representative of three independent transfection experiments. (B) IGV tracks of M-series loci identified from common peaks between live and frozen merged peaks. Tracks are shown as follows (top to bottom): gene models, RNA-seq (WT and differentiated PGCs), ATAC-seq (merged live PGCs, merged frozen PGCs), H3K27ac CUT&Tag and somatic tissue ATAC-tracks. Scale bars indicate 1 kb. Transcripts corresponding to long non-coding RNAs are labelled as "lncRNA" above the respective tracks. Genomic coordinates for each peak and their nearest promoters are listed in Supplementary Table 7.

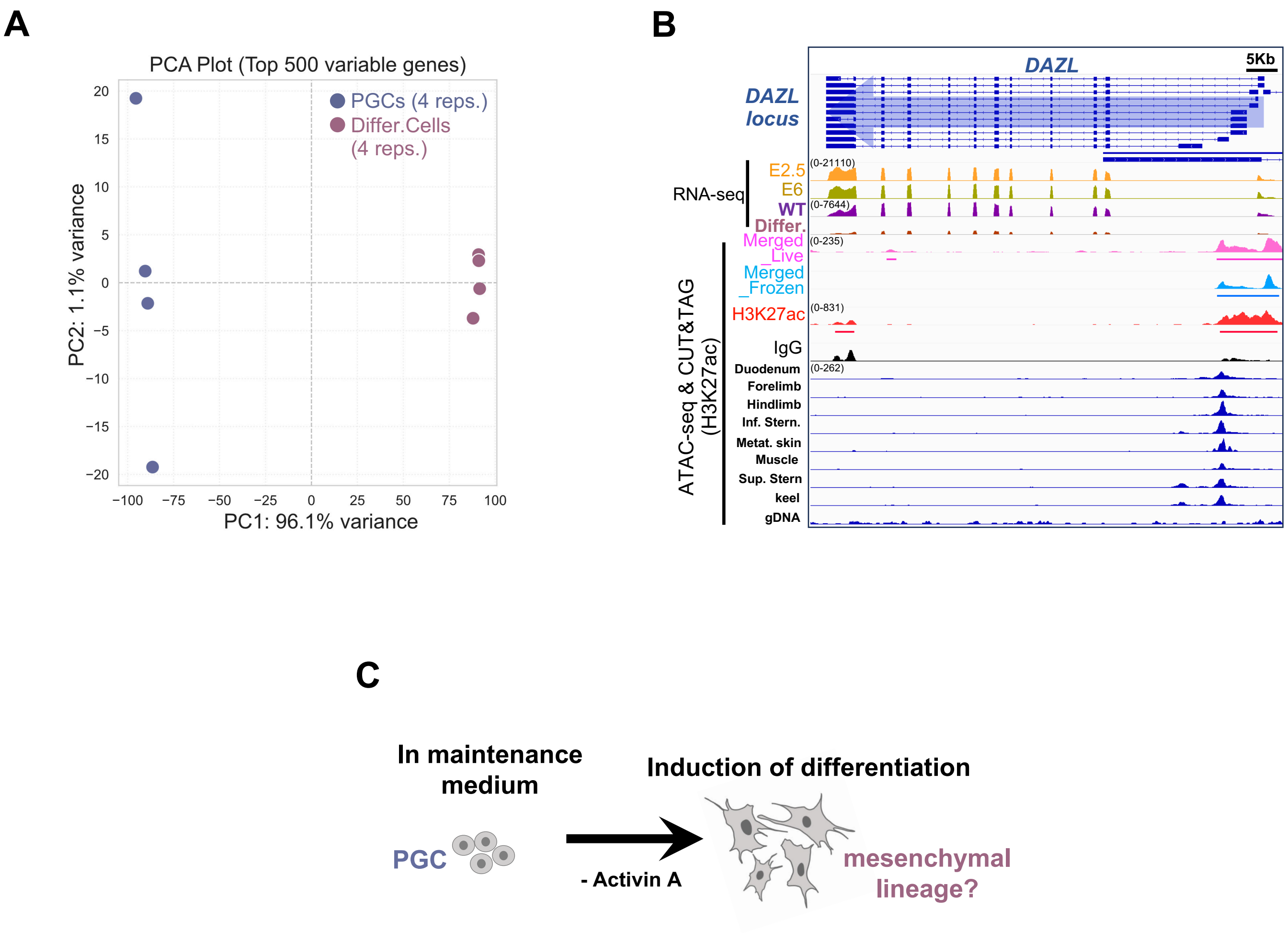

Fig. S6. Transcriptomic analysis of the novel PGC *in vitro* differentiation systems.

(A) Principal Component Analysis (PCA) of transcriptomic profiles from WT PGCs and differentiated cells. PC1 and PC2 account for 96.1% and 1.1% of the total variance, respectively. (B) IGV tracks at *DAZL* locus. Tracks are shown as follows (top to bottom): gene models, RNA-seq (E2.5 and E6, WT PGC and differentiated cells), ATAC-seq (merged live PGCs, merged frozen PGCs), H3K27ac and IgG from CUT&Tag and somatic tissue ATAC-tracks. Scale bar indicates 5 kb. (C) Schematic representation of the predicted differentiation model. The diagram illustrates the cell lineage specification and transitions observed in our *in vitro* differentiation system.

Development • Supplementary information

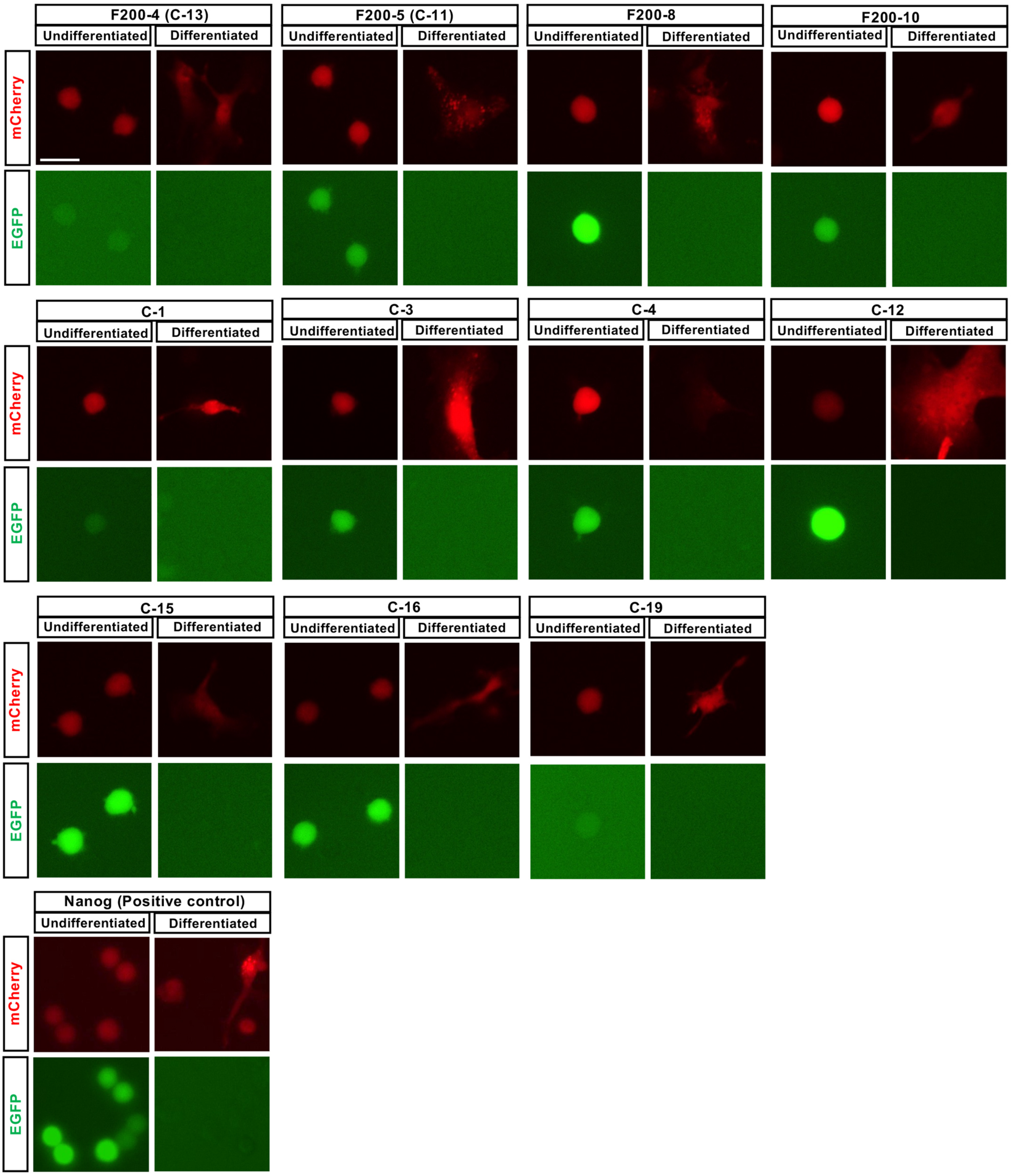

Development • Supplementary information

Fig. S7. ***In vitro*** **differentiation analysis of F200-enhancer candidates.** Cultured PGCs were transfected with individual Tol2-based enhancer–EGFP reporter constructs (F200-series), together with CAGGS-mCherry (transfection control) and CAGGS-T2TP plasmids. Transfected PGCs were maintained under self-renewal conditions (+Activin A) or subjected to *in vitro* differentiation (−Activin A) for 3 weeks. Representative fluorescence images show EGFP and mCherry signals under both conditions. All enhancer constructs that exhibited EGFP fluorescence under maintenance conditions showed reduced or undetectable EGFP expression following differentiation, whereas mCherry fluorescence remained detectable across both conditions. Scale bars: 25 µm. Images are representative of three independent experiments.

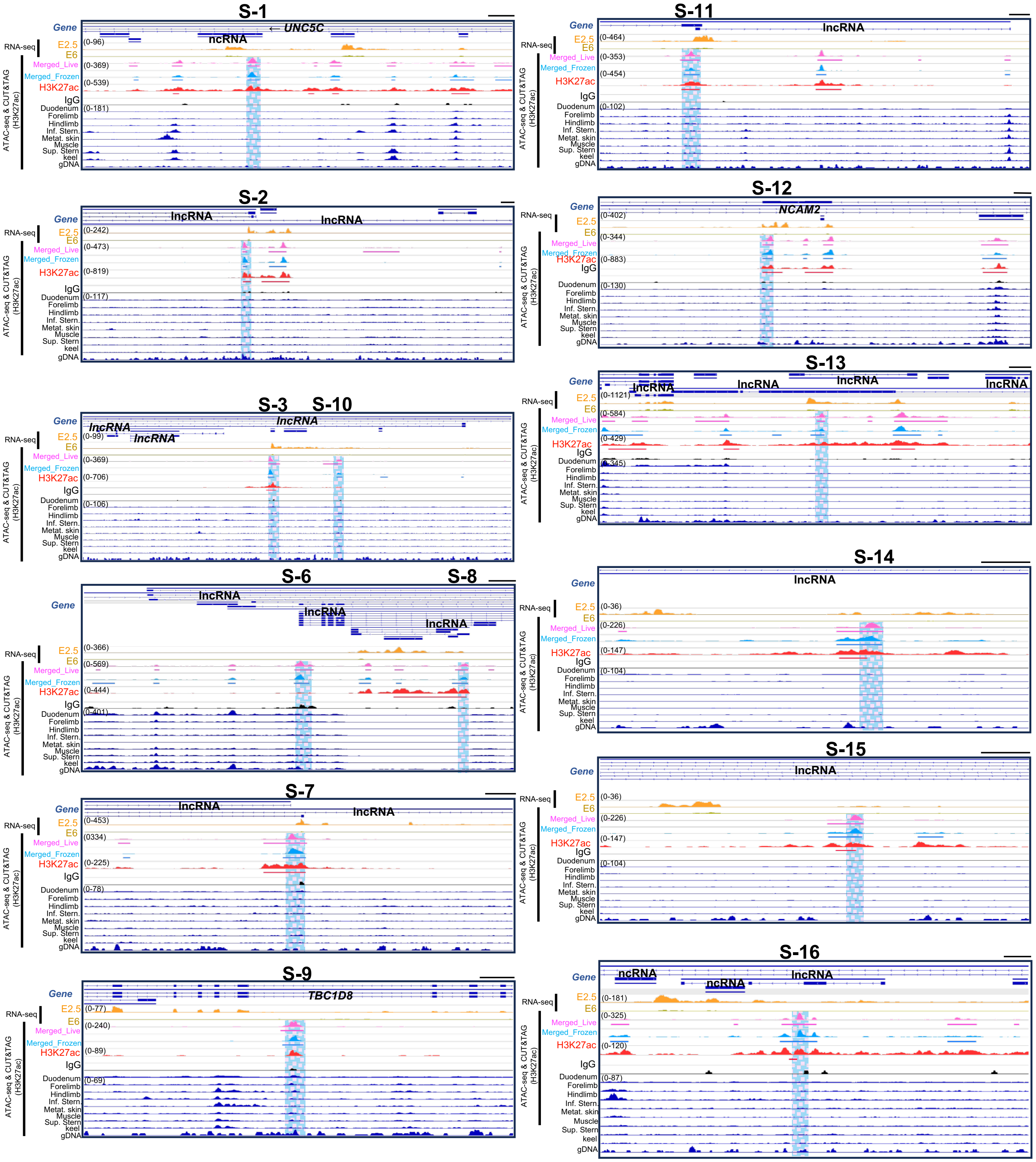

Development • Supplementary information

Fig. S8. Genome tracks of S-series candidate enhancer fragments in cultured PGCs. IGV tracks of S-series loci. Tracks are shown as follows (top to bottom): gene models, RNA-seq (E2.5 and E6), ATAC-seq (merged live PGCs, merged frozen PGCs, and merged somatic tissues), and H3K27ac and IgG CUT&Tag signals. Scale bars represent 1 kb. Transcripts corresponding to long non-coding RNAs are labeled as "lncRNA" above the respective tracks. Genomic coordinates for each peak and their nearest promoters are listed in Table S7. Tracks for S-4 and S-5 are shown in Fig. S10.

Development • Supplementary information

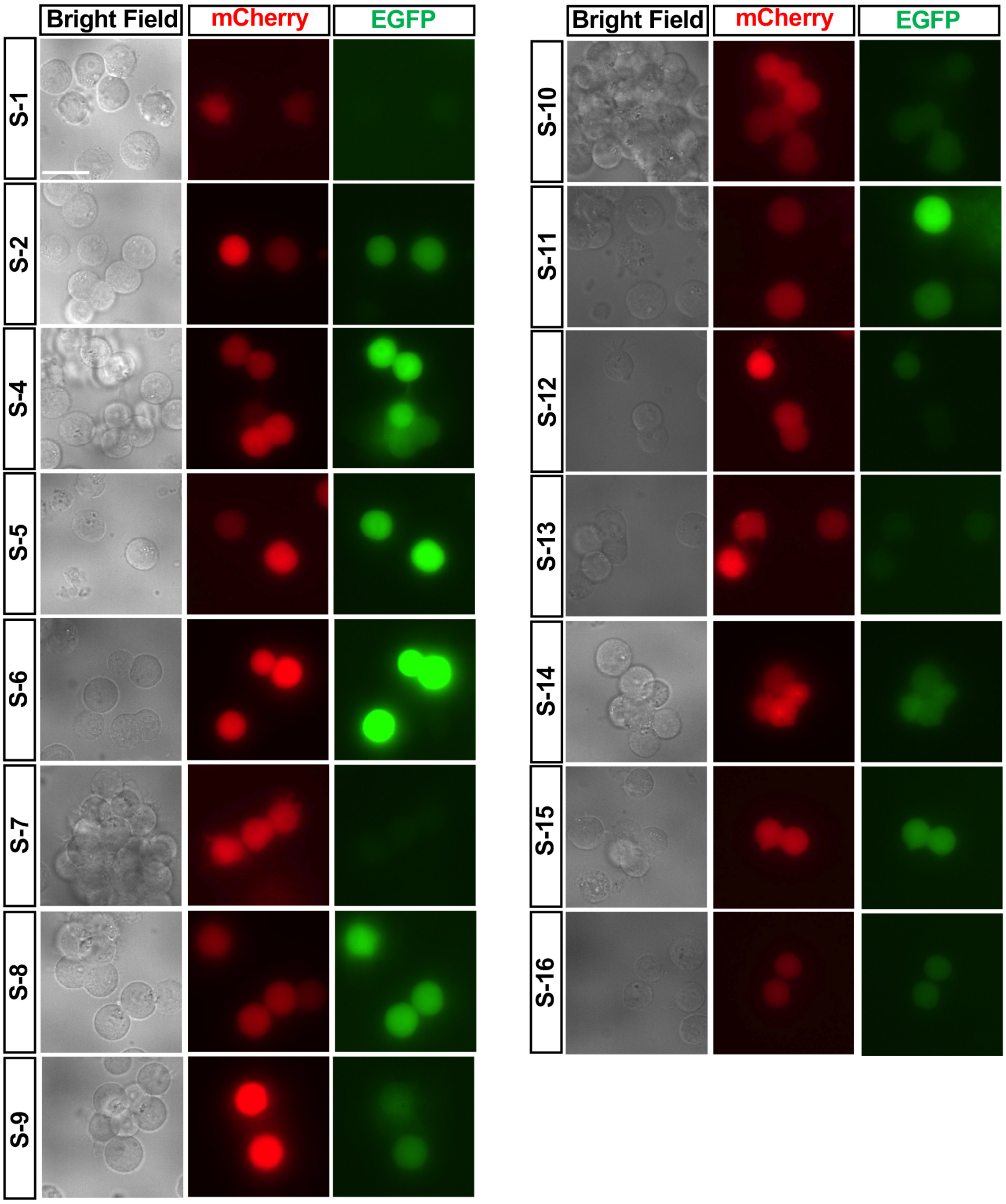

Fig. S9. *In vitro* screening of S-series candidate enhancer fragments in cultured PGCs. Representative fluorescence images of cultured PGCs transfected with individual S-series genomic fragments cloned upstream of a minimal tk promoter driving EGFP. PGCs were co-transfected with enhancer–EGFP reporters, CAGGS-mCherry (transfection control), and CAGGS-T2TP plasmids. Images were acquired under maintenance conditions (+Activin A). For each construct, bright-field, mCherry, and EGFP channels are shown. Scale bars: 25 μm. Images are representative of three independent transfection experiments.

Development • Supplementary information

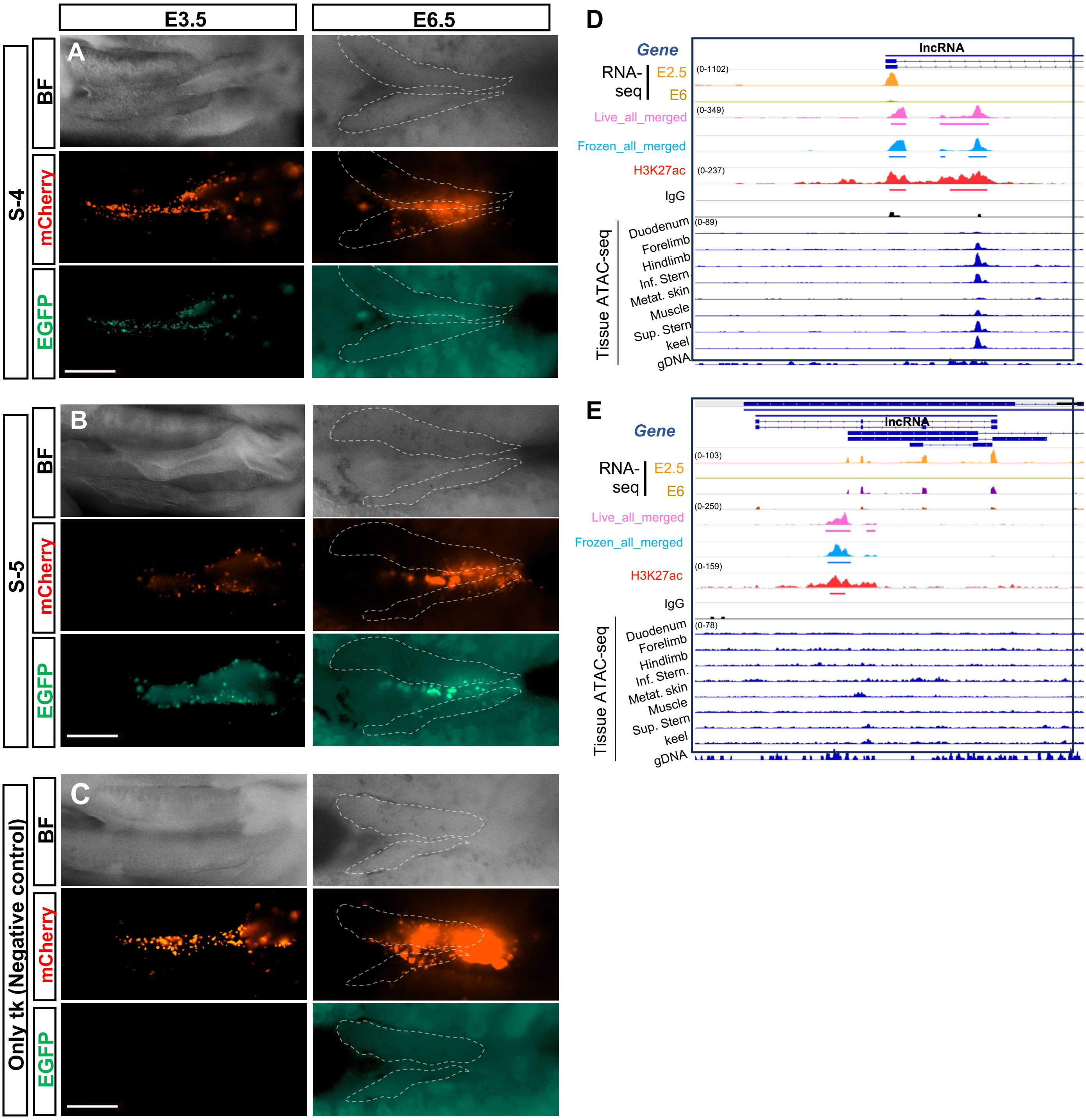

Development • Supplementary information

Fig. S10. ***In vivo transplantation*** **analysis of S-series enhancer candidates.** (A-C) Cultured PGCs were transfected with S-series enhancer-EGFP reporter constructs of S- 4, and S-5, and tk- as a negative control are shown (A), (B), (C), respectively, together with CAGGS-mCherry (transfection control) and CAGGS-T2TP plasmids. Transfected PGCs were maintained under self-renewal conditions (+Activin A) or subjected to *in vitro* differentiation (−Activin A) for 3 weeks. Representative fluorescence images show EGFP and mCherry signals under both conditions. All three S-series enhancer constructs exhibited detectable EGFP fluorescence under maintenance conditions, which was lost following differentiation, whereas mCherry fluorescence remained detectable. Scale bars: 500 µm. Images are representative of three independent experiments. (D-E) IGV tracks of S-series enhancer loci (S-4 and S-5) are shown as follows (top to bottom): gene models, RNA-seq (E2.5 and E6 PGCs), ATAC-seq (merged live PGCs, merged frozen PGCs), H3K27ac CUT&Tag and somatic tissue ATAC-tracks. Scale bars indicate 1 kb. Transcripts corresponding to long non-coding RNAs are labeled as "lncRNA" above the respective tracks. Genomic coordinates for each peak and their nearest promoters are listed in Supplementary Table 7.

Development • Supplementary information

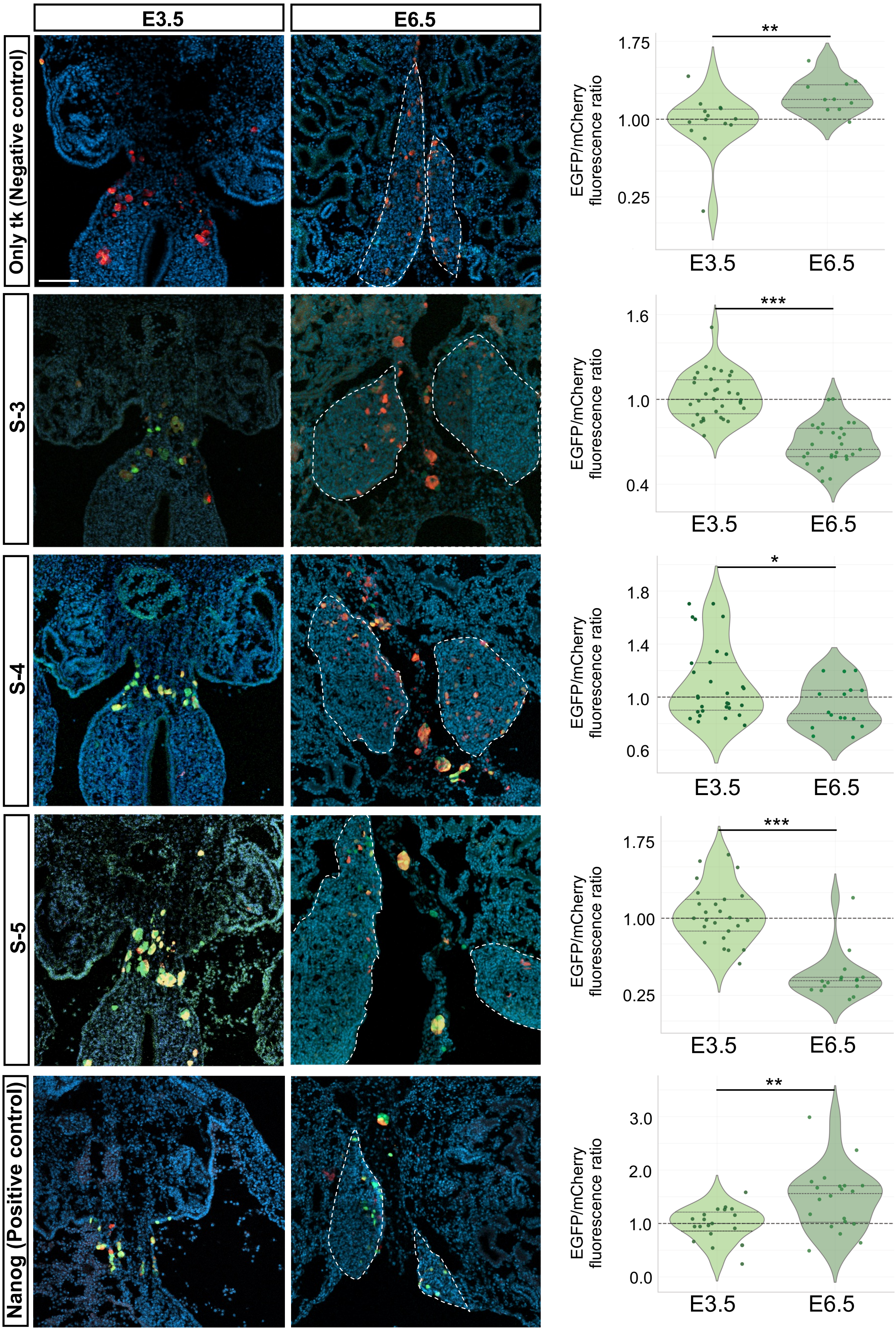
E3.5
E6.5
Only tk (Negative control)
S-3
S-4
S-5
Nanog (Positive control)
EGFP/mCherry fluorescence ratio
**
***
*
***
**
1.75
1.00
0.25
1.6
1.0
0.4
1.8
1.4
1.0
0.6
3.0
2.0
1.0
0.0
E3.5
E6.5

Fig. S11. Immunofluorescence analysis and quantitative assessment of transplanted PGCs *in vivo*.

Representative cryosections of host embryos at E3.5 and E6.5 following transplantation of enhancer reporter-expressing PGCs. Sections were immunostained for EGFP and mCherry, with nuclei counterstained with DAPI. Samples shown include Only tk (negative control), S-3, S-4, S-5, and Nanog promoter-EGFP (positive control). White dotted lines indicate the outline of the developing gonads. Scale bars: 100 µm. All experiments were performed with n=3 embryos per condition. Right panels show quantitative analysis of EGFP fluorescence intensity normalized to mCherry signal, presented as violin plots for individual fields of view. Each dot represents one analyzed region. Statistical comparisons were performed as indicated in the plots.

Table S1. Details of ATAC-seq libraries prepared in this study

Available for download at
https://journals.biologists.com/dev/article-lookup/doi/10.1242/dev.205214#supplementary-data

Table S2. SRA data for ATAC-seq details and accession numbers utilized in the analysis

Available for download at
https://journals.biologists.com/dev/article-lookup/doi/10.1242/dev.205214#supplementary-data

Table S3. Annotated peak features using HOMER annotatePeaks.pl Supplementary

Available for download at
https://journals.biologists.com/dev/article-lookup/doi/10.1242/dev.205214#supplementary-data

Table S4. Genomic location of PGC-specific ACRs

Available for download at
https://journals.biologists.com/dev/article-lookup/doi/10.1242/dev.205214#supplementary-data

Table S5. The gene lists which are assigned nearest promoter of PGC-specific ACRs

Available for download at
https://journals.biologists.com/dev/article-lookup/doi/10.1242/dev.205214#supplementary-data

Table S6. The list of Gene Ontology (GO) results using the gene sets from

Available for download at
https://journals.biologists.com/dev/article-lookup/doi/10.1242/dev.205214#supplementary-data

Table S7. ACR information validated *in vivo* and *in vitro*

Available for download at
https://journals.biologists.com/dev/article-lookup/doi/10.1242/dev.205214#supplementary-data

Table S8. Genomic location of F-200 series of enhancers and their meanFold changes between F200 replicates

Available for download at
https://journals.biologists.com/dev/article-lookup/doi/10.1242/dev.205214#supplementary-data

Table S9. Peak information of peaks from H3K27ac signals

Available for download at
https://journals.biologists.com/dev/article-lookup/doi/10.1242/dev.205214#supplementary-data

Development • Supplementary information

Table S10. Differentially expressed genes in WT_PGC vs Differ_cells by *in vitro* differentiation systems

Available for download at
https://journals.biologists.com/dev/article-lookup/doi/10.1242/dev.205214#supplementary-data

Table S11. Differentially expressed genes in E2.5 vs E6 *in vivo* PGCs Supplementary

Available for download at
https://journals.biologists.com/dev/article-lookup/doi/10.1242/dev.205214#supplementary-data

Table S12. Identification of PGC-specific ACRs associated with E2-high genes

Available for download at
https://journals.biologists.com/dev/article-lookup/doi/10.1242/dev.205214#supplementary-data
