# Supplementary material for: ATAC-seq of low-input and cryopreserved primordial germ cells reveals functional enhancers
Source: Development. 2026 May 12;153(9):dev205214. doi: 10.1242/dev.205214 (PMC13245901; doi:10.1242/dev.205214)
Supplement: Supplementary file 1 [file develop-153-205214_review_history.pdf]

ATAC-seq of low-input and cryopreserved primordial germ cells reveals functional enhancers

Akane Kawaguchi, Mao Igari, Yasuto Murayama, Hiroko Iikawa, Mika Sakamoto, Yasukazu Nakamura, Shigehiro Kuraku, Keisuke Ishihara, Daisuke Saito
DOI: 10.1242/dev.205214

Editor: Haruhiko Koseki

Review timeline

Original submission:	2 September 2025
Editorial decision:	3 October 2025
First revision received:	13 March 2026
Accepted:	7 April 2026

Original submission

First decision letter

MS ID#: dev.205214

MS TITLE: ATAC-seq of Low-Input and Cryopreserved Primordial Germ Cells Reveals Functional Enhancers

AUTHORS: Akane Kawaguchi, Mao Igari, Yasuto Murayama, Hiroko Iikawa, Mika Sakamoto, Yasukazu Nakamura, Shigehiro Kuraku, Keisuke Ishihara and Daisuke Saito

Dear Dr Kawaguchi,

I have now received all the referees reports on the above manuscript, and have reached a decision. The referees' comments are appended below, or you can access them online: please go to . I need to express my apology to have excess reviewers in this case. This is due to technical issue at our side.

The overall evaluation is positive and we would like to publish a revised manuscript in Development, provided that the referees' comments can be satisfactorily addressed. Please attend to all of the reviewers' comments in your revised manuscript and detail them in your point-by-point response. As you see in the reviewer 1's comments, I agree that technical innovation in this study could be limited and better give emphasis on biological aspect. If you do not agree with any of their criticisms or suggestions explain clearly why this is so. If it would be helpful, you are welcome to contact us to discuss your revision in greater detail. Please send us a point-by-point response indicating your plans for addressing the referees' comments, and we will look over this and provide further guidance.

Reviewer 1

Advance summary and potential significance to field

This manuscript describes the use of low-input ATAC-seq to identify active enhancers in cultured chicken primordial germ cells (PGCs). The authors not only report open chromatin regions but also functionally validate a subset of candidate enhancers through reporter assays in PGCs and

© 2026. Published by The Company of Biologists under the terms of the Creative Commons Attribution License (https://creativecommons.org/licenses/by/4.0/).

demonstrate stage-specific activity loss upon differentiation. These biological findings are undoubtedly valuable and will be of interest to researchers working on avian germline development.

However, I do not believe this manuscript meets the standards for publication in Development in its current form, primarily because the central claim — that the authors have established an optimized ATAC-seq method for rare cell types — is not adequately substantiated. The following are my main concerns:

1. Lack of Novelty in the ATAC-seq Protocol
The authors appear to have applied an existing Omni-ATAC protocol to chicken PGCs without any clear technical modifications or innovations. Simply demonstrating feasibility in a specific cell type (PGCs) is not sufficient to support the claim of having "established" or "optimized" a novel method. Furthermore, since the study is limited to a single cell type, the generalizability and broad utility of the method remain unclear. A demonstration in at least one additional rare cell type would be needed to support the broader claims.

2. Logical Issues in Enhancer Candidate Selection
There are conceptual issues in how accessible chromatin regions (ACRs) were selected for enhancer validation:

* First, the authors focus their functional validation on ACRs that are shared between low-cell-number samples (200 cells) and high-input samples (25,000 cells). If the purpose is to validate the utility of ATAC-seq in low-input contexts, then enhancer candidates should be selected from low-input-specific ACRs. Moreover, the study should also assess the biological relevance of ACRs that are only detected in high-input but not in low-input conditions, as these may reflect sensitivity limitations of the method.
* Second, the authors excluded ACRs shorter than 50 bp and longer than 550 bp when selecting enhancer candidates. Unless they provide evidence that such regions do not possess enhancer activity, this exclusion appears arbitrary and potentially overlooks functional elements. A justification or experimental validation of this filtering criterion is warranted.

3. Limited Technical Impact
Overall, while the biological insights regarding chicken PGC-specific regulatory elements are potentially useful, the manuscript does not provide sufficient methodological innovation or validation to support its positioning as a technical advance. As such, it falls short of the impact and rigor expected for publication in Development, particularly in the context of methodological papers.

I recommend rejection of the current version. However, I encourage the authors to consider restructuring the manuscript as a biology-focused study (with less emphasis on technical innovation), or to substantially revise the work by including additional rare cell types and more rigorous benchmarking of their ATAC-seq method.

Reviewer 2

Advance summary and potential significance to field

This manuscript presents a carefully optimized ATAC-seq protocol for profiling chromatin accessibility in cultured chicken primordial germ cells (PGCs), including low-input and cryopreserved samples. The authors successfully demonstrate that high-quality chromatin accessibility profiles can be generated from as few as 200 cells, even after cryopreservation, overcoming a major technical limitation in studying rare cell populations.
A significant strength of the study lies in its integrative approach, combining ATAC-seq data with RNA-seq and tissue-specific datasets to identify more than 10,000 PGC-specific accessible chromatin regions (ACRs). The authors further provide functional validation using reporter assays in cultured PGCs and in vivo transplantation experiments, convincingly showing that many of these ACRs act as enhancers in a stage-dependent manner. The observation that enhancer activity is robust in migratory PGCs but silenced upon gonadal colonization provides new insights into germline-specific regulatory mechanisms. The manuscript is well structured and the results are

© 2026. Published by The Company of Biologists under the terms of the Creative Commons Attribution License (https://creativecommons.org/licenses/by/4.0/).

clearly presented, and the figures effectively illustrate both the technical optimization and biological findings.
Overall, this study makes a valuable contribution to developmental biology and epigenomics by providing both a methodological advance and novel biological insights into germline regulation. However, there are important questions and several points that need revision, and I would like the authors to address these.

Major point
1. Did the authors determine the sex of the embryos before sampling PGCs? In the Methods section, there is no description of sexing, and in all the results and figures the sex of the samples is not indicated. Circulating PGCs at E2.5 show sex-specific differences in gene expression between ZZ and ZW. In particular, genes derived from the Z or W chromosomes may differ in expression and regulation. Please specify the sex of the PGCs used in this study.
2. If sexing was not performed and the analyses were conducted on pooled ZZ and ZW PGCs, would it be experimentally feasible to perform sexing and analyze ZZ and ZW PGCs separately? Alternatively, please explain why it would not be problematic to analyze the samples without distinguishing their sex.
3. Please consider adding the chromosomal location information of the live-specific peaks, cryopreserved-specific peaks, and common peaks in a supplementary table, and illustrating in a figure the distribution trends of peaks mapped to autosomes (macro- and microchromosomes) and sex chromosomes (Z and W). These are expected to provide highly valuable information.
4. In Figure 4E-G, to check the location of fluorescent signals on the gonads, bright-field images are required. Additionally, the images should capture the entire gonad.
5. Related to the previous comment, in the gonads of E6.5 embryos, the distribution of differentiated germ cells (identified by mCherry expression) on the gonads differs between males (ZZ) and females (ZW). Wouldn't sexing of the embryo here as well allow for a more accurate interpretation of the dynamics of the transplanted cells? In the gonads of E6.5 embryos, the right one is degenerated, so sex can also be determined based on morphology. (Is that why the image of the left gonad is shown for E6.5 in Fig. 4D?)

Minor point
6. Line 106-107: Could the authors provide a more detailed explanation of why freeze-thaw steps might lead to increased redundant amplification?
7. Line 111-112: Could you provide an explanation for why mitochondrial removal during lysis is less efficient in cryopreserved cells?
8. For the gonad illustration in Fig. 4D, please either orient it vertically or indicate the dorsal and ventral sides.
9. The file for Supplementary Table 2 is missing, and there are two files labeled as Supplementary Table 4. Please check the table numbering.

Reviewer 3

Advance summary and potential significance to field

Kawaguchi et al. performed bulk ATAC-seq using as few as 200 live or frozen chicken primordial germ cells (PGC). Using this approach, they identified ~10,000 PGC-specific accessible chromatin regions. They then analyzed these ACRs by bioinformatics and tested a subset of them for their activity in reporter assays in chick PGC cultures. They identified 11 of 14 sequences tested that drove reporter expression above basal levels. After injection of PGCs with reporter constructs into the vasculature of chick embryos, they found that these reporters were active at E3.5 but not E6.5.

ATAC-seq studies on rare cell types is challenging. Single cell ATAC-seq can be very expensive. The authors present convincing data on bulk ATAC-seq on very few cells. They also present a pipeline to identify PGC-specific regulatory sequences for their activity in vivo. The findings presented in this paper should be of great interest to readers of Development.

Minor comments:

© 2026. Published by The Company of Biologists under the terms of the Creative Commons Attribution License (https://creativecommons.org/licenses/by/4.0/).

When talking about Tn5 transposase, please do not abbreviate it as Tn5 because it gets confusing whether you are talking about the transposon or transposase. Please use the term Tn5 transposase for the enzyme.
Line 72, perhaps insert text to help the reader: During development, chick PGCs circulate in the blood between stages xx and xx. PGCs isolated from embryonic blood can be cultured and expanded…
Line 74, "endogenous route" may be better to say blood circulation.
Line 187, "We next asked….
Line 348, Banbanker freezing medium?
Line 250, "thrown" should be thawed?
Line 354, provide source of protease inhibitor.
Line 364, "assembled" perhaps synthesized?
Line 366, vortexing
Line 371, "deserved number"? word choice
Line 376, Were both ends sequenced?
Line 517, provide more details about "injected into host chicken embryos". How injected? Injected into which blood vessel? Which stage injected?

Reviewer 4

Advance summary and potential significance to field

The main interest of the paper is that it demonstrates that the ATAC-seq approach can be performed with very few cells, both fresh and frozen. The validation approach is also original and powerful, despite the drawback of not characterizing the induced cells, which may hinder the identification of other regulatory elements.

Comments for the author

In the paper entitled « ATAC-seq of Low-Input and Cryopreserved Primordial Germ Cells Reveals Functional Enhancers», the authors, Kawaguchi et al., identify germ cell specific enhancers through an optimized and elegant ATAC-seq protocol using very few chicken PGCs.
As a general comment, The manuscript is very clear and presents very interesting results on both protocol optimisation and the validation of enhancer sequences specific to germ cells.
There are only a few points that the authors need to address.
Line 60: 'They originate outside the embryo proper': The sentence is ambiguous: PGCs do indeed originate from the embryo! To be reworded.
Lines 105 - 110 - 124: Frozen cells have higher rates of duplication and mtDNA contamination than fresh cells. Although this does not appear to affect library quality, these differences raise questions. The thawing process is not specific in terms of lysis vs. thawing. How much time elapses after thawing, as indicated by the trypan blue count in line 99? Could this be a source of vari-ation, a"nd could the methodology be clarified?
Line 122: "In the end, after removing duplicate reads and mtDNA-mapped reads", It would still be interesting to mention the proportion that was removed and see if there is a bias between the analyses of "fresh" and "thawed" cells. How deep into the analysis is this change? Thank you to the authors for providing this data.
Lines- 129-136: line 138 : This notion of bias and then standardizing the content to 10 million does pose a problem. As mentioned below, would it be possible to do a comparative analysis of the two approaches?
Line 152: Nanog locus: The NANOG gene/locus is not really specific to germ cells alone, as its invalidation does not alter the emergence of the lineage! It would be good if the authors could provide an explanation for this choice. Why not a gene exclusive to the germ line DAZL, CVH, DND, etc… ?
Thank you for the authors' comments.
Lines159-: The observation of a bias in the distribution of Tn5 insertion enriched in intergenic regions is certainly well explained, based on the specificity of the chicken genome architecture (negative control), but the two paragraphs (lines 159-172 and 173-184) are very redundant. The authors could simplify the explanation.

© 2026. Published by The Company of Biologists under the terms of the Creative Commons Attribution License (https://creativecommons.org/licenses/by/4.0/).

To define PGCs—Specific ACRS—Fig. 3 and 3D in particular, the screen compares PGCs with different somatic tissues. While this approach is logical, the difficulty lies in comparing a "purified" cell type, even one maintained in vitro culture, with tissues consisting of a complex set of different cell types. It is therefore difficult to compare the two sets—cells and tissues—especially since the induction of PGC differentiation to validate the elements is not directed toward a particular cell type/lineage. There may therefore be a bias in comparing a cell type with a tissue. Can the authors comment on this? Using a few somatic cell types rather than 'tissues' could undoubtedly lead to the same situation, but it would be more appropriate to compare in vitro models with each other. Could the culture process also modify the distribution of peaks at the level of the identified genes? Can some lineage markers be detected during differentiation induction to get an idea of whether the cells are more mesoderm, endoderm, or neurectoderm—hence the need to include cell references from these lineages in the analysis?
Finally, the validation test for PGC-specific enhancers is particularly elegant, even if it is again difficult to draw conclusions about the nature of cells after their induction into differentiation.
In conclusion, the manuscript is very comprehensive and convincing despite a few comments. Overall, it also demonstrates the feasibility of identifying regulatory elements from very few cells, despite the difficulty of biases observed between fresh and frozen cells.

<u>Reviewer 5</u>

Advance summary and potential significance to field

This manuscript describes the application of Omni-ATAC-seq to study chromatin accessibility dynamics in primordial germ cells (PGCs). Examining DNA accessibility in PGCs is technically challenging due to the fragile nature of the cells and the limited amount of material that can be isolated from the animal. The authors carefully assess the sensitivity of the assay and attempt to validate accessible chromatin regions (ACRs) that appear specifically in cultured chicken PGCs. Both in vitro and in vivo assays are used to demonstrate that the tested ACRs are functional and properly regulated in PGCs. While the method itself is not new, the findings are interesting and merit publication if the following specific questions are adequately addressed.

Comments for the author

Major:
1. One major question is what fraction of the accessible chromatin regions (ACRs) are truly functional. Epigenetically, functionality is not defined by accessibility alone; rather, activity is more accurately indicated by H3K27ac marking or Ep300 binding at the accessible regions. It would therefore be useful to compare Ep300 or H3K27ac peaks in PGCs with the current ATAC-seq data. If such data are not available or cannot be obtained, this limitation should be stated clearly in the text. In that case, I also suggest testing a subset of systematically selected ACRs, perhaps based on rank ordering of accessibility or another criterion, by ChIP-qPCR. Based on the authors' findings, the 14 selected ACRs are likely to be Ep300-bound and H3K27ac marked.
2. I am confused about how to interpret Figure 1I. This figure is not referenced in the text. It seems that live and frozen samples are segregated, and that the frozen 25,000-cell sample aligns well with live samples across different cell numbers. Please include an interpretation of these data.
3. The selection of the 16 ACRs is still unclear. Specify how many peaks passed each filtering step and how these 16 were ultimately chosen.
4. All ACRs should be linked to the nearest genes, and test whether these genes are preferentially expressed in PGCs using transcriptomic data and/or perform GO analysis to determine whether they are enriched for PGC-related terms. While nearest-gene analysis is imperfect, it would still provide valuable insight.
5. In Suppl Fig 3, most ACRs are not linked to known genes. Is this due to limited chicken genome annotation? If so, it might be useful to compare synteny with other genomes to identify orthologous gene names of these, as the current identifiers are not informative.
6. Fig 3E: After activin removal, PGCs adhered and differentiated. What cell types do they differentiate into? Since this is a new finding, it would be useful to clarify the cell type(s).
7. The reporter assays with ACR-EGFP and CAGGS-mCherry are informative but lack quantification. Please report reproducibility across the five injections and provide percentage correlation data for mCherry/EGFP expression (e.g., Suppl. Fig. 2). Similar semi-quantitative analysis should be applied to other datasets.

© 2026. Published by The Company of Biologists under the terms of the Creative Commons Attribution License (https://creativecommons.org/licenses/by/4.0/).

Minor:
1. Figure legends should preferably be written in the present tense, not the past tense. For example, in line 552: "Figure 1 Schema showed…" would be better as "Figure 1 Schema shows…".
2. Gene names in Figures 2G, 1H, and 4B are too small to read.
3. Figure 3H: What is the gene INRAGAL00000002296? The shaded peak area also appears off-center.
4. Figure 4C: The identifiers LOC107055200 and LDHA are not informative without functional context. What is the full spelling of LDHA? Are these genes specifically expressed in PGCs?
5. Suppl Fig 1J: Please clarify how motifs are ordered (e.g., motif rank vs. e-value).
6. Suppl Fig 6: Gene names are missing. The purpose of including the 200-, 1,000-, and 5,000-cell ATAC-seq tracks is unclear. Please clarify whether the frozen-merged profile represents a sum of these datasets, as the live-merged profile in the lower panel more closely resembles the smaller frozen input samples rather than the frozen-merged sample.

First revision

Author response to reviewers' comments

Point-by-point responses to the reviewers' comments

Reviewer 1:

This manuscript describes the use of low-input ATAC-seq to identify active enhancers in cultured chicken primordial germ cells (PGCs). The authors not only report open chromatin regions but also functionally validate a subset of candidate enhancers through reporter assays in PGCs and demonstrate stage-specific activity loss upon differentiation. These biological findings are undoubtedly valuable and will be of interest to researchers working on avian germline development.

However, I do not believe this manuscript meets the standards for publication in Development in its current form, primarily because the central claim — that the authors have established an optimized ATAC-seq method for rare cell types — is not adequately substantiated. The following are my main concerns:

- We thank Reviewer #1 for their careful reading of the manuscript and for recognizing the value of our biological findings regarding PGC-specific regulatory elements and their stage-dependent regulation.

1. Lack of Novelty in the ATAC-seq Protocol

The authors appear to have applied an existing Omni-ATAC protocol to chicken PGCs without any clear technical modifications or innovations. Simply demonstrating feasibility in a specific cell type (PGCs) is not sufficient to support the claim of having "established" or "optimized" a novel method. Furthermore, since the study is limited to a single cell type, the generalizability and broad utility of the method remain unclear. A demonstration in at least one additional rare cell type would be needed to support the broader claims.

- We thank Reviewer #1 for this careful and constructive evaluation. We fully agree that the current manuscript does not introduce a novel ATAC-seq protocol, nor does it include technical modifications to the OMNI-ATAC method, and we acknowledge that language suggesting that we had “established” or “optimized” a new methodology was inappropriate.
Our original intention was not to claim methodological novelty, but rather to address a practical question relevant to many developmental and stem cell systems: whether an established ATAC-seq protocol can be applied under highly constrained experimental conditions, specifically, very small

© 2026. Published by The Company of Biologists under the terms of the Creative Commons Attribution License (https://creativecommons.org/licenses/by/4.0/).

numbers of cryopreserved cells, and still yield biologically interpretable and functionally meaningful results. We chose chicken PGCs as a model because they represent a rare, developmentally well-defined cell population for which downstream functional validation of regulatory elements is experimentally feasible both *in vitro* and *in vivo*.
We recognize that analyses limited to a single cell type cannot support broad claims of methodological generalizability. Accordingly, we have revised the manuscript to remove any implication that this study establishes or optimizes a broadly applicable ATAC-seq method. Instead, we now frame the technical component as an application- and benchmarking-oriented use of an existing protocol under stringent conditions, and we place primary emphasis on the biological outcomes, namely, the identification and functional validation of stage-specific regulatory elements in PGCs.
We believe that this revised framing more accurately reflects the scope and intent of the study and allows the work to be evaluated on its biological contributions rather than on claims of technical innovation.

2. Logical Issues in Enhancer Candidate Selection

There are conceptual issues in how accessible chromatin regions (ACRs) were selected for enhancer validation:
*** First, the authors focus their functional validation on ACRs that are shared between low-cell-number samples (200 cells) and high-input samples (25,000 cells). If the purpose is to validate the utility of ATAC-seq in low-input contexts, then enhancer candidates should be selected from low- input-specific ACRs. Moreover, the study should also assess the biological relevance of ACRs that are only detected in high-input but not in low-input conditions, as these may reflect sensitivity limitations of the method.**

- Thank you very much for this valuable and constructive comment. We agree that, if the goal is to evaluate the utility of ATAC-seq under low-input conditions, functional validation of ACRs identified specifically in low-input samples is particularly important.
In the original analysis, we prioritized ACRs shared between low-cell-number and high-input samples in order to establish a conservative and robust set of candidate enhancers with high reproducibility across conditions. This strategy was intended to minimize false positives when initially defining PGC- specific regulatory elements. However, we acknowledge that this approach alone does not directly address the reviewer's concern regarding the biological relevance of low-input-specific ACRs.
To directly address this point, we have now performed additional analyses focusing exclusively on low- cell-number (Frozen 200 cells), cryopreserved PGC samples. Specifically, we selected the top 14 ACRs ranked by MACS3 (q-value and fold-changes) under this low-input, frozen condition and examined their activity using reporter assays. As a result, enhancer activity was detected in 11 of these 14 ACRs (Figure 3), demonstrating that functionally active enhancers can indeed be identified directly from low- input-specific ACRs.
We conducted differentiation induction experiments to test whether the activity of these enhancers is lost upon in vitro differentiation. Regarding the experimental setup of our in vitro differentiation system, which is described for first time in this study, we conducted RNA-seq to compare WT PGCs and differentiated cells (Supplementary Figure 10). Our result showed that *DAZL*, *Sall4* and *PRDM14* genes which are key signatures of germline stemness were downregulated in the differentiated cells, while mesenchymal marker genes were upregulated. Although extensive analysis of the mechanistic aspect of this differentiation is ongoing, we believe these additional analyses directly address the reviewer's concern and further support the biological relevance of ACRs identified under low-input conditions.

Regarding the biological relevance of ACRs detected only in high-input (25,000 cells) but not in low-input (200 cells) conditions:

We agree that understanding the nature of these "high-input-only" ACRs is crucial for assessing the sensitivity and limitations of our low-input protocol. Since similar comment was given also from reviewer #5, we performed CUT&Tag for H3K27ac. In the result, we found that ACRs in Frozen 200 cell dataset are overlaped with H3K27ac peaks (4/16 in F-200, Figure 4). Regarding the functional nature of these ACRs, we are currently generating a more comprehensive epigenetic landscape that includes H3K4me1, H3K4me3, and H3K9ac. While these datasets are not yet at the

© 2026. Published by The Company of Biologists under the terms of the Creative Commons Attribution License (https://creativecommons.org/licenses/by/4.0/).

publicable scale (e.g., number of replicates) required for a full genome-wide publication, we found that the other detected ACRs are also marked with active histone mark (H3K4me3; 9/16 in F-200, Unpublished data). Consequently, we reason that our low-input ATAC-seq protocol effectively captures the most robust and functionally relevant regulatory elements by acting as a stringent filter against regions with marginal activity. We included detailed discussion on this sensitivity-threshold trade-off and the comparative analysis in the revised manuscript.

*** Second, the authors excluded ACRs shorter than 50 bp and longer than 550 bp when selecting enhancer candidates. Unless they provide evidence that such regions do not possess enhancer activity, this exclusion appears arbitrary and potentially overlooks functional elements. A justification or experimental validation of this filtering criterion is warranted.**

- Thank you for this thoughtful comment. We agree that length-based filtering of ACRs requires careful justification and should be clearly explained.
In our initial analysis, we applied a size filter (50–550 bp) primarily as a practical criterion to prioritize ACRs that are technically amenable to cloning and functional validation using reporter assays. Very short ACRs often yield weak or inconsistent reporter signals, whereas very long regions frequently represent repetitive elements, such as simple repeat elements, complicating both cloning procedures and functional interpretation. Importantly, this filtering was not intended to imply that ACRs outside this size range lack enhancer potential, but rather to enable systematic and interpretable experimental validation within the scope of the present study.
To more directly address the reviewer's concern, we have now performed additional functional analyses focusing on ACRs identified specifically from the low-cell-number (200 cells), cryopreserved PGC dataset. We selected the top-ranked ACRs based on accessibility signal under this condition and assessed their enhancer activity using reporter assays. The majority of these regions exhibited clear enhancer activity, providing experimental support that our selection strategy robustly captures biologically meaningful regulatory elements even under low-input, frozen conditions.
We have revised the manuscript to clarify both the rationale and the limitations of this size-based filtering and to avoid overinterpretation of this criterion.

3. Limited Technical Impact

Overall, while the biological insights regarding chicken PGC-specific regulatory elements are potentially useful, the manuscript does not provide sufficient methodological innovation or validation to support its positioning as a technical advance. As such, it falls short of the impact and rigor expected for publication in Development, particularly in the context of methodological papers.
- We thank the reviewer for this important and candid assessment. We agree that the present study does not aim to introduce a novel ATAC-seq methodology or to constitute a major technical advance, and we fully acknowledge that it should not be positioned as a methodological paper.
The primary objective of this work was to examine the practical applicability and limitations of an existing OMNI-ATAC-seq protocol when applied to an experimentally constrained biological system— specifically, very small numbers of cryopreserved chicken primordial germ cells—and to use the resulting chromatin accessibility data to identify and functionally validate PGC-specific and stage- dependent regulatory elements. Accordingly, the methodological component of this study is intended as an application- and benchmarking-oriented use of an established technique, rather than as a source of technical innovation.
From this perspective, we believe that the value of the study lies not in methodological novelty, but in demonstrating, through functional validation, that biologically meaningful and lineage-specific enhancers can be recovered from low-input, frozen samples. This practical demonstration may be informative for researchers working with rare or experimentally challenging cell populations under similar constraints.
To better reflect this scope, we have revised the manuscript to remove any implication that it represents a technical advance, to moderate methodological claims throughout, and to place primary emphasis on the biological findings and their relevance to germline development.

I recommend rejection of the current version. However, I encourage the authors to consider restructuring the manuscript as a biology-focused study (with less emphasis on technical

© 2026. Published by The Company of Biologists under the terms of the Creative Commons Attribution License (https://creativecommons.org/licenses/by/4.0/).

innovation), or to substantially revise the work by including additional rare cell types and more rigorous benchmarking of their ATAC-seq method.

We thank the reviewer for this frank and constructive recommendation. We fully understand the rationale for recommending rejection of the current version and appreciate the clear guidance regarding possible directions for revision.
- In line with the reviewer's suggestion, we have chosen to substantially restructure the manuscript as a biology-focused study, rather than to pursue a methodological expansion involving additional rare cell types. Accordingly, we have revised the manuscript to markedly reduce the emphasis on technical innovation and benchmarking as a primary contribution, and instead to focus on the biological insights gained from chromatin accessibility analysis and functional validation of stage-specific regulatory elements in chick PGCs.
Specifically, we have revised the abstract, introduction, and main text to remove claims of methodological advancement, to frame the ATAC-seq analyses as an application of an existing protocol under experimentally constrained conditions, and to emphasize the identification, validation, and developmental regulation of PGC-specific enhancers. Additional functional analyses derived from low- input, cryopreserved samples have been incorporated to strengthen this biological narrative.
While we acknowledge that inclusion of additional rare cell types would be necessary to support broader methodological generalization, we believe that such an expansion would constitute a distinct technical study beyond the scope of the present work. We therefore aim for this revised manuscript to be evaluated primarily on its biological contributions to the understanding of germline regulatory mechanisms.

Reviewer 2:

This manuscript presents a carefully optimized ATAC-seq protocol for profiling chromatin accessibility in cultured chicken primordial germ cells (PGCs), including low-input and cryopreserved samples. The authors successfully demonstrate that high-quality chromatin accessibility profiles can be generated from as few as 200 cells, even after cryopreservation, overcoming a major technical limitation in studying rare cell populations.
A significant strength of the study lies in its integrative approach, combining ATAC-seq data with RNA-seq and tissue-specific datasets to identify more than 10,000 PGC-specific accessible chromatin regions (ACRs). The authors further provide functional validation using reporter assays in cultured PGCs and *in vivo* transplantation experiments, convincingly showing that many of these ACRs act as enhancers in a stage-dependent manner. The observation that enhancer activity is robust in migratory PGCs but silenced upon gonadal colonization provides new insights into germline-specific regulatory mechanisms. The manuscript is well structured and the results are clearly presented, and the figures effectively illustrate both the technical optimization and biological findings.
Overall, this study makes a valuable contribution to developmental biology and epigenomics by providing both a methodological advance and novel biological insights into germline regulation. However, there are important questions and several points that need revision, and I would like the authors to address these.
- We thank the reviewer for this positive and constructive evaluation of our manuscript. We appreciate the recognition of both the technical aspects of the low-input ATAC-seq analysis and the biological insights into PGC-specific, stage-dependent regulatory elements. We have carefully addressed the points raised and revised the manuscript accordingly to improve clarity and rigor.

Major point

1. Did the authors determine the sex of the embryos before sampling PGCs? In the Methods section, there is no description of sexing, and in all the results and figures the sex of the samples is not indicated. Circulating PGCs at E2.5 show sex-specific differences in gene expression between ZZ and ZW. In particular, genes derived from the Z or W chromosomes may differ in expression and regulation. Please specify the sex of the PGCs used in this study.

- We apologize for the lack of information regarding the sex of the samples in the original manuscript. We agree with the reviewer that this is an important point. All PGCs used in this study were derived from female (ZW) embryos, and this information has now been explicitly added

© 2026. Published by The Company of Biologists under the terms of the Creative Commons Attribution License (https://creativecommons.org/licenses/by/4.0/).

to the Methods section (Line 383).

2. If sexing was not performed and the analyses were conducted on pooled ZZ and ZW PGCs, would it be experimentally feasible to perform sexing and analyse ZZ and ZW PGCs separately? Alternatively, please explain why it would not be problematic to analyse the samples without distinguishing their sex.

- We fully agree with the reviewer that sex differences can influence gene expression and regulatory mechanisms in chicken PGCs, particularly for Z- and W-linked genes, and that this is an important consideration when interpreting data at E2.5.

For the ATAC-seq analyses in this study, all samples were derived from female (ZW) PGCs, and therefore are not affected by sex mixing. This point has now been clarified in the Methods section (Line 383). For the RNA-seq analyses, we incorporated previously published datasets from Ichikawa et al., which include both male (ZZ) and female (ZW) PGCs (two biological replicates each per stage). This dataset was selected to ensure sufficient biological replication for robust statistical comparisons between developmental stages (E2.5 and E6.0), rather than to specifically address sex-dependent gene regulation. Accordingly, our primary analytical focus was on identifying developmental stage-specific regulatory elements, rather than resolving sex-specific differences in cis-regulatory mechanisms.

While we agree that separate analyses of ZZ and ZW PGCs would be valuable for dissecting sex-specific regulatory landscapes, such analyses fall outside the scope of the present study. We believe that our integrated approach nevertheless provides valid insights into PGC-specific enhancers, particularly given that the chromatin accessibility data used to define enhancer candidates originate exclusively from ZW-derived PGCs.

To address this limitation explicitly, we have revised the Discussion to acknowledge the potential influence of sex differences and to highlight future directions aimed at investigating sex-specific enhancer usage in PGCs, especially for Z- and W-linked genes (Line 212 - 216).

3. Please consider adding the chromosomal location information of the live-specific peaks, cryopreserved-specific peaks, and common peaks in a supplementary table, and illustrating in a figure the distribution trends of peaks mapped to autosomes (macro- and microchromosomes) and sex chromosomes (Z and W). These are expected to provide highly valuable information.

- We thank the reviewer for this helpful suggestion. As noted, we originally provided a comprehensive list of identified PGC-specific ACRs in Supplementary Table 4. To improve clarity and address the reviewer's comment, we agree that it is more informative to explicitly distinguish peaks by experimental condition.

In addition, in response to the reviewer's suggestion to visualize chromosomal distribution, we have performed additional analyses and will include new supplementary figures illustrating the distribution of ACRs across autosomes (macro- and micro-chromosomes) and sex chromosomes (Z and W). These analyses were conducted separately for commonly detected ACRs between PGC and somatic tissues and PGC-specific ACRs (new Figure 3E, 3F and new Sup. Figure 2).

When normalized as peaks per Mbp, we observed a modest trend in which ACR density was slightly lower on macro-chromosomes (Chr.1-9) and somewhat higher on micro-chromosomes (Chr.10-39) (new Figure 3E, 3F and new Sup. Figure 2). This pattern appeared to track with gene density across chromosomes, suggesting that the subtle macro-micro- difference likely reflects underlying genomic feature density rather than a biological bias.

In contrast, we observed distinct patterns on the sex chromosomes. On the Z chromosome, the density of total detected ACRs and PGC-specific ACRs was 27.95 and 5.39 per Mbp, respectively, whereas on the W chromosome these values were 29.31 and 13.39 per Mbp. These results suggest a potential sex chromosome-associated bias in chromatin accessibility, consistent with the reviewer's comment. We have included these data in the revised supplementary figures and have added a brief discussion noting this observation and its potential implications (Line 212-216), while acknowledging that a detailed analysis of sex-specific regulatory mechanisms is beyond the scope of our study.

4. In Figure 4E-G, to check the location of fluorescent signals on the gonads, bright-field images are required. Additionally, the images should capture the entire gonad.

- We apologize that the original images did not clearly convey the tissue context. To address this concern, we have revised the imaging strategy in new Figures 5E, 5G and new Sup. Figure 10A,

© 2026. Published by The Company of Biologists under the terms of the Creative Commons Attribution License (https://creativecommons.org/licenses/by/4.0/).

10B and 10C. Specifically, we now provide updated whole-mount images that include corresponding bright-field views and capture the entire gonad, allowing clear visualization of the anatomical context.
In addition, to more precisely demonstrate the localization of transplanted PGCs and its relationship to signal intensity, we have prepared and included sectional images. These additions clarify the spatial distribution of fluorescent signals within the gonadal tissue and improve the interpretability of the data.

5. Related to the previous comment, in the gonads of E6.5 embryos, the distribution of differentiated germ cells (identified by mCherry expression) on the gonads differs between males (ZZ) and females (ZW). Wouldn't sexing of the embryo here as well allow for a more accurate interpretation of the dynamics of the transplanted cells? In the gonads of E6.5 embryos, the right one is degenerated, so sex can also be determined based on morphology. (Is that why the image of the left gonad is shown for E6.5 in Fig. 4D?)
- As the reviewer correctly points out, E6.5 represents a developmental stage at which sexual dimorphism in the gonads begins to emerge. In this study, however, the ACRs selected for enhancer validation were associated with genes that show stage-dependent downregulation at E6.5 relative to E2.5 in both male (ZZ) and female (ZW) PGCs. Our analysis was therefore designed to capture developmental stage-specific regulation rather than sex-specific differences.
For the quantitative analyses, we measured fluorescence intensity in PGCs located within both left and right gonads at E6.5 embryos. At this stage, overt degeneration of the right gonad in female embryos has not yet progressed substantially, and we did not observe systematic differences in PGC localization or EGFP/mCherry ratios between the two sides. Thus, no consistent left-right or sex-dependent bias was detected in our dataset.
Taken, together, these considerations suggest that sex-related differences are unlikely to confound the interpretation of stage-dependent enhancer silencing observed at E6.5.

Minor point

6. Line 106-107: Could the authors provide a more detailed explanation of why freeze-thaw steps might lead to increased redundant amplification?
7. Line 111-112: Could you provide an explanation for why mitochondrial removal during lysis is less efficient in cryopreserved cells?
- Thank you for these important questions. While we did not directly investigate the mechanistic basis of reduced mitochondrial removal efficiency in cryopreserved samples, we speculate that cryopreservation may alter cellular membrane properties, potentially affecting lipid composition or membrane integrity. Such changes could reduce the efficiency of detergent-based lysis conditions used in the standard OMNI-ATAC protocol (e.g. Tween-20, Digitonin, and NP-40), leading to incomplete separation of nuclei from surrounding mitochondria.
As a consequence, residual mitochondria may remain associated with isolated nuclei, resulting in increased mitochondrial DNA tagmentation. Because mitochondrial DNA lacks nucleosome organization, it is more readily tagmented, which could in turn contribute to higher duplication rates and altered library complexity in cryopreserved samples compared with live cells.
We emphasize that this explanation is speculative, and that a systematic investigation of membrane properties or protocol optimization would be required to directly address this issue. We have now added a brief discussion of these possibilities and limitations in the revised manuscript (Line 123-130).

Reviewer 3:

Kawaguchi et al. performed bulk ATAC-seq using as few as 200 live or frozen chicken primordial germ cells (PGC). Using this approach, they identified ~10,000 PGC-specific accessible chromatin regions. They then analyzed these ACRs by bioinformatics and tested a subset of them for their activity in reporter assays in chick PGC cultures. They identified 11 of 14 sequences tested that drove reporter expression above basal levels. After injection of PGCs with reporter constructs into the vasculature of chick embryos, they found that these reporters were active at E3.5 but not E6.5.
ATAC-seq studies on rare cell types is challenging. Single cell ATAC-seq can be very expensive. The authors present convincing data on bulk ATAC-seq on very few cells. They also present a pipeline to identify PGC-specific regulatory sequences for their activity *in vivo*.

© 2026. Published by The Company of Biologists under the terms of the Creative Commons Attribution License (https://creativecommons.org/licenses/by/4.0/).

The findings presented in this paper should be of great interest to readers of Development.
- We thank Reviewer #3 for this positive and encouraging evaluation of our work. We appreciate the reviewer's recognition of the technical feasibility of bulk ATAC-seq from very small numbers of cells and of the biological significance of identifying and functionally validating PGC-specific regulatory elements *in vivo*.

Minor comments:

When talking about Tn5 transposase, please do not abbreviate it as Tn5 because it gets confusing whether you are talking about the transposon or transposase. Please use the term Tn5 transposase for the enzyme.
- Thank you for this helpful suggestion. We have revised the manuscript to consistently use the term

"Tn5 transposase" when referring to the enzyme, in order to avoid confusion.

Line 72, perhaps insert text to help the reader: During development, chick PGCs circulate in the blood between stages xx and xx. PGCs isolated from embryonic blood can be cultured and expanded…
Line 74, "endogenous route" may be better to say blood circulation.
Line 187, "We next asked….
Line 348, Banbanker freezing medium?
Line 250, "thrown" should be thawed?
Line 354, provide source of protease inhibitor.
Line 364, "assembled" perhaps synthesized?
Line 366, vortexing
Line 371, "deserved number"? word choice.
Line 376, Were both ends sequenced?
Line 517, provide more details about "injected into host chicken embryos". How injected? Injected into which blood vessel? Which stage injected?
- Thank you for these helpful and detailed suggestions. We have carefully addressed all the points raised, including clarifying the description of PGC circulation and terminology, correcting typographical errors, refining word choices, and providing additional methodological details (e.g. sequencing strategy, injection procedure, and reagent sources). The manuscript has been revised accordingly to improve clarity and accuracy.

Reviewer 4:

SUMMARY OF THE ADVANCE MADE IN THIS PAPER AND ITS POTENTIAL SIGNIFICANCE TO THE FIELD
The main interest of the paper is that it demonstrates that the ATAC-seq approach can be performed with very few cells, both fresh and frozen. The validation approach is also original and powerful, despite the drawback of not characterizing the induced cells, which may hinder the identification of other regulatory elements.
- We thank the reviewer for this clear summary and for recognizing the significance of our study, particularly the applicability of ATAC-seq to very small numbers of fresh and frozen cells and the strength of the functional validation approach.

SUGGESTIONS TO AUTHORS
In the paper entitled « ATAC-seq of Low-Input and Cryopreserved Primordial Germ Cells Reveals Functional Enhancers», the authors, Kawaguchi et al., identify germ cell specific enhancers through an optimized and elegant ATAC-seq protocol using very few chicken PGCs. As a general comment, The manuscript is very clear and presents very interesting results on both protocol optimisation and the validation of enhancer sequences specific to germ cells. There are only a few points that the authors need to address.

Line 60: 'They originate outside the embryo proper': The sentence is ambiguous: PGCs do indeed originate from the embryo! To be reworded.

© 2026. Published by The Company of Biologists under the terms of the Creative Commons Attribution License (https://creativecommons.org/licenses/by/4.0/).

- Thank you for pointing out this ambiguity. We have revised the sentence to clarify this point and now state that “PGCs originate in the peripheral regions of the early embryo” (Line 49-52).

Lines 105 - 110 - 124: Frozen cells have higher rates of duplication and mtDNA contamination than fresh cells. Although this does not appear to affect library quality, these differences raise questions. The thawing process is not specific in terms of lysis vs. thawing. How much time elapses after thawing, as indicated by the trypan blue count in line 99? Could this be a source of vari- ation, a"nd could the methodology be clarified?

- Thank you for this question. As shown in the figure, we performed tripan blue staining and observed that most of the cells were intact (Fig.1B and 1C). Regarding the thawing and lysis timing, the lysis buffer was added to the cells at the same time-line as the tripan blue staining (Line 100-101). Therefore, we believe that cell death and/or cell membrane rupture were not the reason for the high mtDNA contamination in our frozen libraries.

As mentioned above, our speculation on this matter can be found in points 6 and 7 of reviewer #2's questions, and the details can be found there. In brief, we hypothesise that cryopreservation may alter cellular membrane properties, potentially affecting lipid composition or membrane integrity. Such changes could reduce the efficiency of the detergent-based lysis conditions used in the standard OMNI- ATAC protocol, leading to incomplete separation of the nuclei from the surrounding mitochondria. We emphasise that this explanation is speculative and that a systematic investigation of membrane properties or protocol optimisation would be required to address this issue directly. We have now added a brief discussion of these possibilities and limitations in the revised manuscript (Line 123-130).

Line 122: "In the end, after removing duplicate reads and mtDNA-mapped reads", It would still be interesting to mention the proportion that was removed and see if there is a bias between the analyses of "fresh" and "thawed" cells. How deep into the analysis is this change? Thank you to the authors for providing this data.
Thank you for the comment. We prepared Supplementary Table 2-4 that includes the details of quality control and the details of detected ACRs.

Lines- 129-136: line 138 : This notion of bias and then standardizing the content to 10 million does pose a problem. As mentioned below, would it be possible to do a comparative analysis of the two approaches?
- Thank you for asking this important question. We chose to down-sample all libraries to 10 million reads not as a way to test for bias, but as a necessary quality control step to ensure a truly fair comparison. The logic is straightforward. The total read count of our smallest library was approximately 10 million, this became our baseline of the libraries. If we had used the raw, varying read counts, samples with higher depth would naturally show more peaks simply due to higher sensitivity (as implied in Fig. 2A and B). This would create a technical artifact, making it impossible to tell if a difference between Live and Frozen samples was biological or just a result of sequencing depth.

Our data in Figs. 2C-D support this strategy. As shown in Fig. 2C, when standardized to this depth, there is no meaningful difference in peak numbers between the two groups. More importantly, Fig. 2E demonstrates that robust promoter-associated peaks are consistently captured in both conditions. While some weaker, intergenic peaks might fluctuate, the core regulatory landscape remains stable.
In short, 10 million reads provided enough depth to assess the library quality essential for Fig. 1 and 2, while protecting our analysis from the bias of uneven sequencing coverage. We believe this quality assessment approach prioritizes biological accuracy and reinforces the validity of our comparison in the following analysis.

Line 152: Nanog locus: The NANOG gene/locus is not really specific to germ cells alone, as its invalidation does not alter the emergence of the lineage! It would be good if the authors could provide an explanation for this choice. Why not a gene exclusive to the germ line DAZL, CVH, DND, etc… ?
- Thank you for your insightful comment. We focused on the *NANOG* locus throughout our analysis and provided these tracks because we utilized a *NANOG* reporter construct to monitor PGC activity both *in vitro* and *in vivo*. We agree that *NANOG* is not exclusively expressed in the germline. However, it served as a primary marker for our reporter system. As suggested, to

© 2026. Published by The Company of Biologists under the terms of the Creative Commons Attribution License (https://creativecommons.org/licenses/by/4.0/).

demonstrate the consistency of our data with germline specific markers, we have now included the genomic locus for *DAZL*, along with all relevant experimental tracks (transcriptomic data, ATAC-seq peaks, and CUT&Tag peaks), in the new Supplementary Figure 4B.

Lines159-: The observation of a bias in the distribution of Tn5 insertion enriched in intergenic regions is certainly well explained, based on the specificity of the chicken genome architecture (negative control), but the two paragraphs (lines 159-172 and 173-184) are very redundant. The authors could simplify the explanation.
- Thank you for this helpful suggestion. We agree that the two paragraphs were repetitive. In the revised manuscript, we have merged and streamlined the explanation to remove redundancy, while keeping the key message that the intergenic enrichment reflects the chicken genome architecture and is supported by the gDNA-based negative control. The relevant text has been revised accordingly (Line 175-182).

To define PGCs—Specific ACRS—Fig. 3 and 3D in particular, the screen compares PGCs with different somatic tissues. While this approach is logical, the difficulty lies in comparing a "purified" cell type, even one maintained *in vitro* culture, with tissues consisting of a complex set of different cell types. It is therefore difficult to compare the two sets—cells and tissues—especially since the induction of PGC differentiation to validate the elements is not directed toward a particular cell type/lineage. There may therefore be a bias in comparing a cell type with a tissue. Can the authors comment on this? Using a few somatic cell types rather than 'tissues' could undoubtedly lead to the same situation, but it would be more appropriate to compare *in vitro* models with each other. Could the culture process also modify the distribution of peaks at the level of the identified genes?
- We appreciate the reviewer's thoughtful comment regarding the challenge of comparing a purified cell population (cultured PGCs) with heterogeneous somatic tissues. We agree that such comparisons have inherent limitations in terms of cell-type resolution, and that this represents an important conceptual consideration.
Our rationale for using heterogeneous somatic tissues as a reference was based on the goal of defining PGC-enriched ACRs in a stringent manner. Because these tissues comprise a mixture of multiple somatic cell types, regulatory regions that are accessible both in PGCs and across diverse somatic lineages are unlikely to represent germline-specific elements. In this context, the heterogeneity of the somatic tissues serves as a conservative background, allowing us to filter out broadly accessible regulatory regions and to prioritize ACRs enriched specifically in PGCs. In this sense, we consider the complexity of the tissue samples to strengthen, rather than weaken, their utility as a reference.
We acknowledge that comparing *in vitro* PGCs with *in vitro* derived somatic cell types could represent an alternative strategy, and that such comparisons may provide complementary insights. However, we note that cultured somatic cell types, such as DF-1 and DT40 derived chick embryos, also represent selected and artificial states, and thus may introduce different but comparable biases. We have therefore opted to clearly define our approach as a conservative screening strategy rather than a comprehensive resolution of cell-type-specific regulatory landscapes.
Regarding the potential influence of the culture process on chromatin accessibility, we recognize that

in vitro culture conditions may modify the distribution of accessible regions at certain loci. While this possibility cannot be entirely excluded, the fact that the identified ACRs show strong stage-dependent activity *in vivo* supports their biological relevance.

Can some lineage markers be detected during differentiation induction to get an idea of whether the cells are more mesoderm, endoderm, or neurectoderm—hence the need to include cell references from these lineages in the analysis?
Finally, the validation test for PGC-specific enhancers is particularly elegant, even if it is again difficult to draw conclusions about the nature of cells after their induction into differentiation.
In conclusion, the manuscript is very comprehensive and convincing despite a few comments. Overall, it also demonstrates the feasibility of identifying regulatory elements from very few cells, despite the difficulty of biases observed between fresh and frozen cells.

© 2026. Published by The Company of Biologists under the terms of the Creative Commons Attribution License (https://creativecommons.org/licenses/by/4.0/).

- We appreciate the reviewer's valuable suggestion regarding the characterization of lineage identity in differentiated PGCs. We agree that assessing lineage-specific markers would help clarify the cellular state following Activin withdrawal and strengthen the interpretation of the enhancer validation assays. To address this point, we performed RNA-seq analyses of differentiated PGCs (differentiated through Activin A withdrawal) in parallel with cultured PGC (WT PGCs with Activin A). These new data are now presented in new Figure 4 and Supplementary Figure 4. Our RNA-seq results reveal that PGC and pluripotency markers, such as *DAZL*, *NANOG*, and *LIN28A*, are significantly down-regulated, whereas mesoderm markers, such as *POSTN*, *DCN* and *MYH1D*, are up-regulated upon in vitro differentiation (please see in Line 268-282). While we are currently investigating the molecular mechanisms underlying this dynamic lineage transition induced by Activin A withdrawal, our results clearly demonstrate that this system is robust enough to monitor the inactivation of PGC-specific enhancer activities.

We should note that while these analyses provide insight into the general lineage tendencies of the differentiated cells, the differentiation protocol was not designed to drive cells toward a specific somatic lineage. Accordingly, we interpret this assay primarily as a functional tool to assess the exit from the PGC state and the subsequent inactivation of PGC-specific enhancers, rather than an attempt to define fully committed somatic identities.

Reviewer 5:

SUMMARY OF THE ADVANCE MADE IN THIS PAPER AND ITS POTENTIAL SIGNIFICANCE TO THE FIELD

This manuscript describes the application of Omni-ATAC-seq to study chromatin accessibility dynamics in primordial germ cells (PGCs). Examining DNA accessibility in PGCs is technically challenging due to the fragile nature of the cells and the limited amount of material that can be isolated from the animal. The authors carefully assess the sensitivity of the assay and attempt to validate accessible chromatin regions (ACRs) that appear specifically in cultured chicken PGCs. Both *in vitro* and *in vivo* assays are used to demonstrate that the tested ACRs are functional and properly regulated in PGCs. While the method itself is not new, the findings are interesting and merit publication if the following specific questions are adequately addressed.

- We thank Reviewer #5 for this thoughtful summary and for recognizing the technical challenges associated with profiling chromatin accessibility in PGCs, as well as the value of our functional validation approaches *in vitro* and *in vivo*.

SUGGESTIONS TO AUTHORS

Major:

1. One major question is what fraction of the accessible chromatin regions (ACRs) are truly functional. Epigenetically, functionality is not defined by accessibility alone; rather, activity is more accurately indicated by H3K27ac marking or Ep300 binding at the accessible regions. It would therefore be useful to compare Ep300 or H3K27ac peaks in PGCs with the current ATAC- seq data. If such data are not available or cannot be obtained, this limitation should be stated clearly in the text. In that case, I also suggest testing a subset of systematically selected ACRs, perhaps based on rank ordering of accessibility or another criterion, by ChIP-qPCR. Based on the authors' findings, the 14 selected ACRs are likely to be Ep300-bound and H3K27ac marked.

- We agree with Reviewer #5 that chromatin accessibility alone does not fully define enhancer functionality, and that additional epigenetic features such as H3K27ac enrichment or EP300 binding provide stronger evidence of regulatory activity.

At present, high-quality antibodies suitable for ChIP-based detection of avian EP300 are not available, which precludes direct assessment of EP300 occupancy in chicken PGCs. However, we have performed complementary epigenomic profiling in cultured PGCs, including CUT&Tag analysis for H3K27ac. We have now included a subset of these results in the revised manuscript to provide additional epigenetic support for the identified ACRs. Furthermore, to address the reviewer's concern regarding the functional nature of these ACRs, we are currently generating a more comprehensive epigenetic landscape that includes H3K4me1, H3K4me3, and H3K9ac. While these datasets are not yet at the exhaustive scale (e.g., number of replicates) required for a full

© 2026. Published by The Company of Biologists under the terms of the Creative Commons Attribution License (https://creativecommons.org/licenses/by/4.0/).

genome-wide publication, they provide strong preliminary evidence.

For the reviewer's reference only, we would like to provide an IGV track of the *DAZL* locus below, which is a key factor in germline development. This visualization demonstrates that the identified ACRs at this critical locus are robustly marked by H3K27ac, H3K4me3, and H3K9ac, reinforcing our conclusion that the ACRs captured in our Omni-ATAC-seq data represent *bona fide* functional regulatory elements.

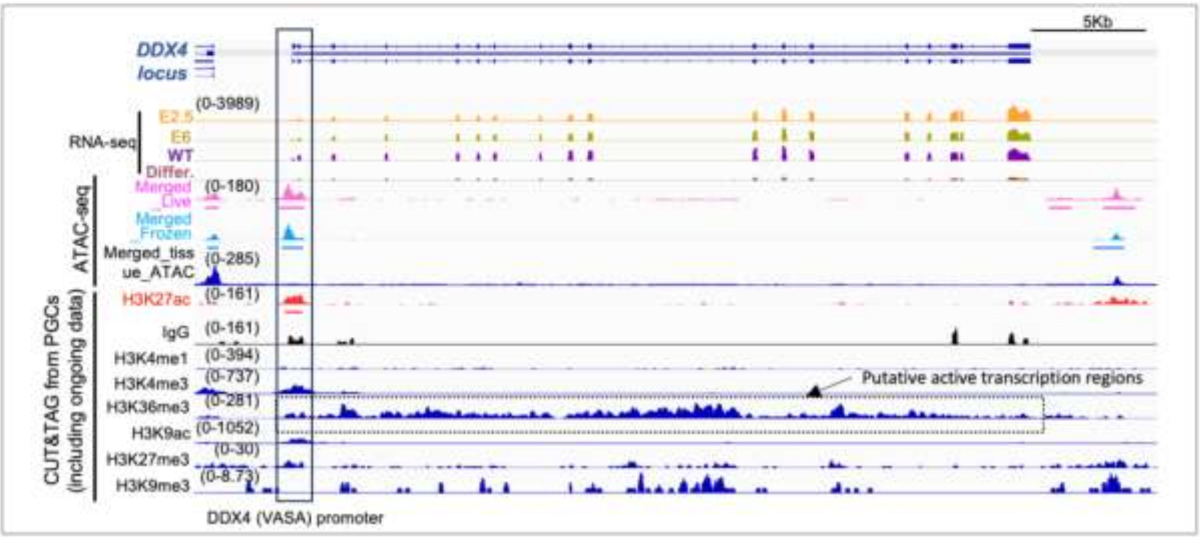

2. I am confused about how to interpret Figure 1I. This figure is not referenced in the text. It seems that live and frozen samples are segregated, and that the frozen 25,000-cell sample aligns well with live samples across different cell numbers. Please include an interpretation of these data.
- Thank you for pointing this out. We apologize for the lack of explanation and for not explicitly referencing Figure 1I in the original text (please see in Line 121-123).
Figure 1I shows a principal component analysis (PCA) of ATAC-seq datasets generated from live and cryopreserved PGC samples across different input cell numbers. This analysis was intended as a quality control assessment to evaluate the overall similarity between libraries. The plot indicates that samples primarily segregate according to live versus frozen conditions, while the 25,000-cell frozen sample clusters closely with live samples across cell numbers, suggesting that cryopreservation does not fundamentally alter global chromatin accessibility profiles when sufficient input material is available.

3. The selection of the 16 ACRs is still unclear. Specify how many peaks passed each filtering step and how these 16 were ultimately chosen.
- Thank you for raising this important point. We agree that the rationale for selecting the initially tested 16 ACRs should be clarified more explicitly.

As stated in our response to Reviewer #1, the central aim of this study is to evaluate whether biologically meaningful regulatory elements can be recovered under experimentally constrained conditions, specifically from very small numbers of cryopreserved cells. In line with this focus, we have revised the manuscript to place primary emphasis on the analysis of the top-ranked ACRs identified from low- input (200-cell), cryopreserved PGC samples, and these data now form the core of the revised study. The originally tested set of 16 ACRs was not selected based on ranking or predicted activity, but rather sampled from a broader pool of candidate regions. We acknowledge that this selection is therefore not systematic and could be considered arbitrary. In the revised manuscript, we have repositioned these data accordingly: rather than presenting them as representative top candidates, we now describe them as demonstrating that functional enhancers can be recovered even from lower-ranked or non-prioritized ACRs identified under our experimental conditions.

By clearly distinguishing between these two analyses, the revised manuscript emphasizes both (i) the robustness of enhancer identification from low-input, frozen samples using top-ranked ACRs,

© 2026. Published by The Company of Biologists under the terms of the Creative Commons Attribution License (https://creativecommons.org/licenses/by/4.0/).

and (ii) the broader feasibility of recovering functional enhancers even when candidate selection is not optimized. We believe this revised framing improves transparency and more accurately reflects the scope and conclusions of the study.

4. All ACRs should be linked to the nearest genes, and test whether these genes are preferentially expressed in PGCs using transcriptomic data and/or perform GO analysis to determine whether they are enriched for PGC-related terms. While nearest-gene analysis is imperfect, it would still provide valuable insight.

- Thank you for this constructive suggestion. We agree that linking accessible chromatin regions (ACRs) to nearby genes and evaluating their transcriptional relevance would provide additional biological insight.

In the revised manuscript, we have associated all identified PGC-specific ACRs (over 10,000 regions) with their nearest genes based on genomic proximity using HOMER's annotatePeaks.pl (now these closest genes are listed in Supplementary Table 5). Regarding GO analysis with the genes of closest PGC-specific ACRs, we performed functional enrichment using g:Profiler. However, we did not find significant enrichment for specific germline- or PGC-related biological process terms (Supplementary Table 6 and we attached the landscapes from overall result, please *see below*). As you noted, we acknowledge that nearest-gene assignment is an imperfect proxy for ACRs to target relationships, particularly for distal regulatory elements that may bypass the nearest promoter to regulate more distant genes.

In addition, we asked if the genes which are highly expressed in E2.5 are located within 2,500bp from PGC-specific ACRs (Supplementary Table12). In the results of the analysis, we found that several key PGC-related genes, such as *LIN28A* and *ZIC3*, which are highly expressed at E2.5, harbour PGC- specific ACRs near their coding regions. Similarly, *ALDH1A1*, which shows high expression in E6
PGCs, appears to be regulated by nearby ACRs located within exonic or intronic regions. We believe this analysis provides a valuable first-order view of the regulatory landscape and supports the association of these ACRs with PGC-specific transcriptional programs.

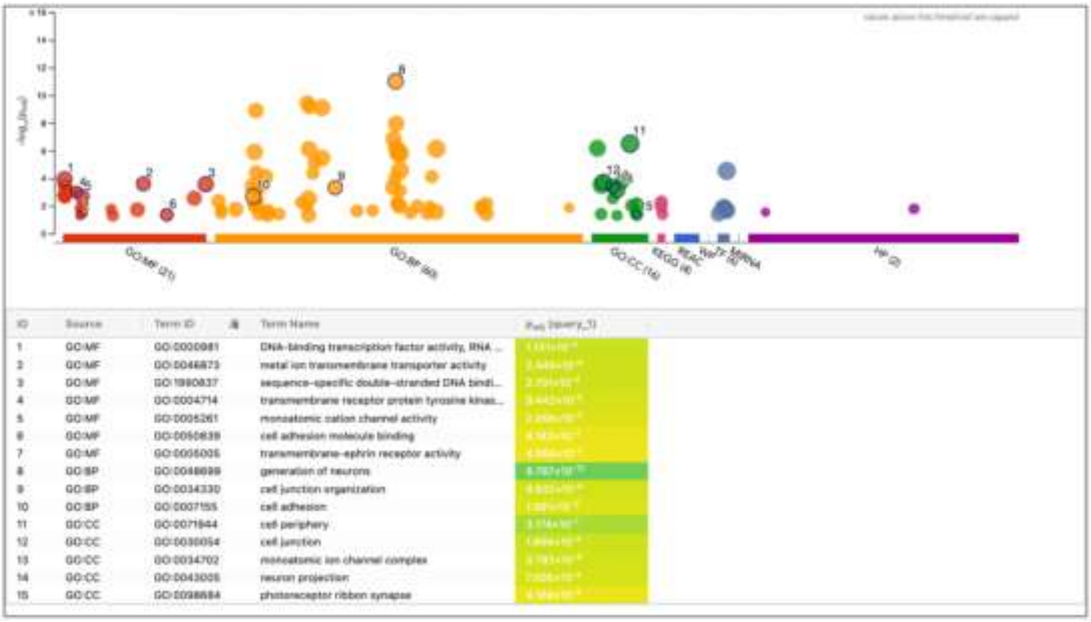

ID	Source	Term ID	Term Name	p_{adj} (query_1)
1	GO:MF	GO:0000981	DNA-binding transcription factor activity, RNA ...	[illegible]
2	GO:MF	GO:0046873	metal ion transmembrane transporter activity	[illegible]
3	GO:MF	GO:1990837	sequence-specific double-stranded DNA bindi...	[illegible]
4	GO:MF	GO:0004714	transmembrane receptor protein tyrosine kinas...	[illegible]
5	GO:MF	GO:0005261	monoatomic cation channel activity	[illegible]
6	GO:MF	GO:0050839	cell adhesion molecule binding	[illegible]
7	GO:MF	GO:0005005	transmembrane-ephrin receptor activity	[illegible]
8	GO:BP	GO:0048699	generation of neurons	[illegible]
9	GO:BP	GO:0034330	cell junction organization	[illegible]
10	GO:BP	GO:0007155	cell adhesion	[illegible]
11	GO:CC	GO:0071944	cell periphery	[illegible]
12	GO:CC	GO:0030054	cell junction	[illegible]
13	GO:CC	GO:0034702	monoatomic ion channel complex	[illegible]
14	GO:CC	GO:0043005	neuron projection	[illegible]
15	GO:CC	GO:0098684	photoreceptor ribbon synapse	[illegible]

5. In Suppl Fig 3, most ACRs are not linked to known genes. Is this due to limited chicken genome annotation? If so, it might be useful to compare synteny with other genomes to identify orthologous gene names of these, as the current identifiers are not informative.

Thank you for this insightful comment regarding the gene annotation. The high proportion of uncharacterized genes or non-coding RNAs in the tracks are primarily a reflection of our decision to use the latest Chick Atlas annotation (bGalGal1.mat.broiler.GRCg7b) (Degalez et al., 2024). We opted for this specific dataset because it integrates an extensive range of novel transcripts, which is derived from RNA-seq across 47 chicken tissues, that were largely absent in previous versions. While this inevitably introduces many "LOC.." or uncharacterized identifiers "NONGGAG..", we felt

© 2026. Published by The Company of Biologists under the terms of the Creative Commons Attribution License (https://creativecommons.org/licenses/by/4.0/).

that relying on more restricted, older annotations would risk overlooking avian-specific regulatory elements that may be critical for PGC development. In our view, providing a comprehensive catalogue of these regions is more valuable for the community than filtering the data down to only well-known protein- coding genes.

We fully agree that identifying orthologous relationships in better-annotated genomes is essential for clarifying the genomic context of these ACRs. To address this, we attempted a manual synteny analysis by comparing the loci with the mouse genome (mm10). Furthermore, we conducted BLAST searches to identify potential orthologous counterparts for these transcripts. However, we found a difficulty to annotated and find the orthologues genes with mouse due to the complex annotation and putative chick specific gene features. At least in the revised version, we added the closest annotation with ACRs to provide information in new Supplementary Tables 5.

6. Fig 3E: After activin removal, PGCs adhered and differentiated. What cell types do they differentiate into? Since this is a new finding, it would be useful to clarify the cell type(s).

- We thank the reviewer for this insightful comment. In this study, the differentiation condition achieved by Activin withdrawal was primarily used to establish a “non-PGC” state, enabling us to test whether PGC-specific enhancers are selectively inactivated upon loss of PGC identity. Our main objective was therefore not to direct differentiation toward a specific somatic lineage, but to assess the lineage specificity of the identified regulatory elements.

While the precise identity of the differentiated cells has not yet been fully defined, they exhibit clear and reproducible morphological changes, including increased adherence and altered cell shape (Fig. 4), consistent with a transition away from the PGC state. In addition, our in vivo experiments support this interpretation, as the PGC-specific enhancers identified in this study naturally lose their activity between E3.5 and E6.0 during normal embryonic development.

As we answered to Reviser#4, we performed RNA-seq analyses of differentiated cells in parallel with cultured PGC. These new data are now presented in new Figure 4 and Supplementary Figure 4. Our RNA-seq results reveal that PGC and pluripotency markers, such as *DAZL, NANOG*, and *LIN28A*, are significantly down-regulated, whereas mesoderm markers, such as *POSTN, DCN* and *MYH1D* (please see in Line 268-282), are up-regulated upon in vitro differentiation. While we are currently investigating the molecular mechanisms underlying this dynamic lineage transition induced by Activin A withdrawal, our results clearly demonstrate that this system is robust enough to monitor the inactivation of PGC- specific enhancer activities.

7. The reporter assays with ACR-EGFP and CAGGS-mCherry are informative but lack quantification. Please report reproducibility across the five injections and provide percentage correlation data for mCherry/EGFP expression (e.g., Suppl. Fig. 2). Similar semi-quantitative analysis should be applied to other datasets.

- Thank you for this important suggestion. We agree that quantitative assessment is essential to support the reproducibility and robustness of the reporter assays.

To address this point, we have generated serial sections of transplanted embryos and performed quantitative fluorescence analysis at the single-cell level. Specifically, for each transplanted PGC, we measured EGFP and mCherry fluorescence intensities and calculated the EGFP/mCherry ratio across multiple sections and embryos derived from independent injections. This approach allowed us to assess both reproducibility across the three injections and the correlation between enhancer-driven EGFP expression and the internal CAGGS-mCherry control.

We have now incorporated these semi-quantitative analyses into the revised manuscript and corresponding Supplementary Figure 11, and we have applied the same quantification strategy to additional reporter datasets where applicable. These data provide quantitative support for the conclusions drawn from the reporter assays.

Minor:

1. Figure legends should preferably be written in the present tense, not the past tense. For example, in line 552: "Figure 1 Schema showed…" would be better as "Figure 1 Schema shows…".
2. Gene names in Figures 2G, 1H, and 4B are too small to read.

- We apologize for the legibility issues regarding the gene labels in the original figures. Following

© 2026. Published by The Company of Biologists under the terms of the Creative Commons Attribution License (https://creativecommons.org/licenses/by/4.0/).

your suggestion, we have increased the font size for the gene symbols in Figures 2G, 2H, and 5B to improve readability. Specifically, for the heatmap in Figure 5B, the high density of gene features including many uncharacterized transcripts from the latest Chick Atlas annotation presented a challenge for labelling. To correct this, we manually curated the labels in the established gene symbols are now displayed at 8-point font, while less characterized or long identifiers are set at 5-point font to maintain clarity without overcrowding the plot. Furthermore, we have ensured that all DEGs are comprehensively listed in Supplementary Table 11 for the readers' reference. We believe these modifications significantly enhance the clarity of the figures while maintaining the necessary genomic detail.

3. Figure 3H: What is the gene INRAGAL00000002296? The shaded peak area also appears off- center.
- Regarding the identity of INRAGAL00000002296, this is a long non-coding RNA (lncRNA) newly identified by the Chick Atlas project. It is located on Chromosome 1 (approximately 127.47-127.62 Mb) in a region largely devoid of protein-coding genes. In fact, our manual inspection of the genomic neighbourhood revealed only uncharacterized loci (e.g., *LOC100315695* and *LOC121107417*), suggesting that this area represents a novel avian-specific regulatory landscape. In the revised version, the figure order and context have been sightly changed. We levelled those as “ncRNA” or “IncRNA” when we found the uncharacterized lncRNA features. Regarding the shaded peak area in Figure 3H, we apologize for the visual misalignment. We have corrected the figure in the revised manuscript to ensure the shaded region is precisely centred over the peak of the ATAC-seq signals.

4. Figure 4C: The identifiers LOC107055200 and LDHA are not informative without functional context. What is the full spelling of LDHA? Are these genes specifically expressed in PGCs?
- Thank you for the opportunity to clarify the functional context of these genes. In the previous version, we highlighted *LOC107055200* locus which are highly expressed in E2.5 PGC. In the revised version we replaced the panel at *ERNI* locus (Eraly Neural Induction gene) which is also highly expressed in E2.5 (See also volcano plot in Fig.5B). Moreover, *LDHA* stands for Lactate Dehydrogenase A. We identified *LDHA* as a Differentially Expressed Gene (DEG) that exhibits low expression at E2.5 but becomes significantly upregulated by E6 (Fig. 5 volcano plot, and Supplementary table 11). This up-regulation timing coincides with the period when PGCs have colonized the at E6 gonad and undergo active proliferation. The upregulation of *LDHA* may suggests a metabolic shift toward aerobic glycolysis (often associated with rapid cell proliferation and pluripotency maintenance) as PGCs transition from their migratory phase to the gonadal environment. In the revised version, we have updated the Figure legend and the Results section to include the full name of *LDHA*.

5. Suppl Fig 1J: Please clarify how motifs are ordered (e.g., motif rank vs. e-value).
- Regarding the ordering of motifs in Supplementary Figure 1J, we have updated the figure legend to explicitly state that the motifs are ranked based on their MEME-assigned E-value (Line 828-829). MEME (MEME suite (Motif-based sequence analysis tools: https://meme-suite.org/meme/db/motifs)) identifies conserved motifs within a set of sequences by evaluating their statistical significance. The E- value represents the expected number of motifs of the same width and occurrence that would be found in a random sequence set of the same size. Accordingly, the motifs are presented in order of their statistical significance, with the top-ranked motifs possessing the lowest E-values.

6. Suppl Fig 6: Gene names are missing. The purpose of including the 200-, 1,000-, and 5,000-cell ATAC-seq tracks is unclear. Please clarify whether the frozen-merged profile represents a sum of these datasets, as the live-merged profile in the lower panel more closely resembles the smaller frozen input samples rather than the frozen-merged sample.

- Thank you very much for this helpful comment. The intention of this panel is to evaluate the biological relevance of the S-series candidate ACRs through integration of multiple datasets. Specifically, ATAC- seq tracks from live and cryopreserved PGC samples are shown as merged profiles to allow direct comparison of chromatin accessibility patterns between the two conditions. The consistency of these profiles supports the robustness of the identified ACRs across experimental conditions. In addition, H3K27ac CUT&Tag data are included to indicate that these regions correspond to active chromatin states consistent with enhancer activity. RNA-seq data

© 2026. Published by The Company of Biologists under the terms of the Creative Commons Attribution License (https://creativecommons.org/licenses/by/4.0/).

comparing PGCs and differentiated cells are also shown to examine whether the nearby genes exhibit PGC-state-dependent expression patterns. Furthermore, publicly available ATAC-seq datasets from multiple somatic tissues are displayed for comparison, allowing assessment of whether these ACRs are specific to PGCs. Thus, this figure was designed to demonstrate that the S-series ACRs are likely to represent biologically relevant regulatory elements by showing that they (1) are accessible specifically in PGCs, (2) coincide with active chromatin marks, and (3) are located near genes whose expression patterns are consistent with PGC identity. These points have now been clarified in the revised figure legends.

Second decision letter

MS ID#: dev.205214R1

MS TITLE: ATAC-seq of Low-Input and Cryopreserved Primordial Germ Cells Reveals Functional Enhancers

AUTHORS: Akane Kawaguchi, Mao Igari, Yasuto Murayama, Hiroko Iikawa, Mika Sakamoto, Yasukazu Nakamura, Shigehiro Kuraku, Keisuke Ishihara and Daisuke Saito

Dear Dr Kawaguchi,

I am happy to tell you that your manuscript has been accepted for publication in Development, pending our standard publication integrity checks.

Reviewer 2

Advance summary and potential significance to field

The authors have responded appropriately to my comments, and I have confirmed the revisions to the manuscript and figures.

Reviewer 3

Advance summary and potential significance to field

I stand with my previous comments on the manuscript. This is an important contribution that many readers of Development will find interesting and useful.

Comments for the author

I believe the authors have more than adequately addressed the initial reviews.

Reviewer 4

Advance summary and potential significance to field

The authors have essentially addressed all the questions I raised, particularly by including data on the DAZL locus and clarifying the nuances involved in the differentiation process. I find this very satisfying.

Reviewer 5

© 2026. Published by The Company of Biologists under the terms of the Creative Commons Attribution License (https://creativecommons.org/licenses/by/4.0/).

Advance summary and potential significance to field

The authors have addressed all of the concerns I previously raised in a satisfactory manner. This manuscript makes important technical and biological contributions by describing not only a novel ATAC-seq approach for cryopreserved samples, but also the changes in DNA accessibility that occur during primordial germ cell formation in the chick. Furthermore, the authors demonstrate that many of these accessible regions are functional and act as early-stage enhancers in PGCs. Overall, the ability to identify candidate enhancers from such a limited number of cells is particularly noteworthy and will be of broad interest to researchers working with rare cell types. This is an excellent and compelling study, and I am very pleased to recommend it for publication.

Comments for the author

S-5 in Supple Figure 8 is missing.

© 2026. Published by The Company of Biologists under the terms of the Creative Commons Attribution License (https://creativecommons.org/licenses/by/4.0/).
